# An advancement in developmental and reproductive toxicity (DART) risk assessment: evaluation of a bioactivity and exposure-based NAM toolbox

**DOI:** 10.3389/ftox.2025.1602065

**Published:** 2025-06-30

**Authors:** Iris Mueller, Ashraf Abdelkhaliq, Paul Carmichael, Matthew Dent, Marleen Feliksik, Luke Flatt, Jade Houghton, José M. Horcas Nieto, Amer Jamalpoor, Predrag Kukic, Sophie Malcomber, Beate Nicol, Gopal Pawar, Claire Peart, Katarzyna Przybylak, Magdalena Sawicka, Katy Wilson, Kathryn Wolton

**Affiliations:** ^1^ Unilever, Sharnbrook, United Kingdom; ^2^ Toxys, Oegstgeest, Netherlands

**Keywords:** new approach methodologies, risk assessment, developmental and reproductive toxicity, bioactivity, exposure, *in silico*

## Abstract

Traditional chemical safety assessment involves identifying the lowest level of a chemical that impacts endpoints measured in standardized animal studies to establish human exposure limits. *In vitro* assays have shown promise in providing points of departure that can be protective of human health when combined with exposure predictions into a bioactivity:exposure ratio (BER). Using a combination of broad screening tools and DART-targeted assays, we previously demonstrated high biological coverage of this NAM toolbox against a list of DART-relevant genes and pathways. To fully transition to an animal-free paradigm, it is crucial to establish confidence that these *in vitro* assays sufficiently represent the DART toxicity mechanisms, ensuring a level of protection that is safe for non-pregnant adults, pregnant women, and fetal populations. In this proof-of-concept study, we have extended the toolbox to include additional *in vitro* and *in silico* tools and have performed an evaluation using 37 benchmark compounds across 49 exposure scenarios. According to existing regulatory opinions, 18 of these scenarios would be considered high-risk chemical exposures from a DART perspective. Our DART NAM toolbox approach identified 17 out of these 18 high-risk scenarios. We further investigated the impact of population-based changes in pregnancy and the fetus on internal exposures by evaluating human clinical data where available for the 37 compounds. In most instances, the variability resulting from pregnancy or gestational changes falls within the range of toxicokinetic variability observed in the general population. This work demonstrates that protective safety decisions can be made for DART without generating new animal test data.

## 1 Introduction

Significant progress has been made in adopting New Approach Methodologies (NAMs) for chemical safety assessment. NAMs have been particularly successful for local toxicity endpoints like skin corrosion, eye damage, and skin sensitization (Sewell et al., 2024). To allow safety assessment of chemicals NAMs will also be needed for more complex endpoints. To this end, Next-Generation Risk Assessment (NGRA) approaches are increasingly being developed (Thomas et al., 2019; Berggren et al., 2017). These approaches are exposure-led, and hypothesis driven, using a tiered, iterative approach to make safety decisions, designed to prevent harm (Dent et al., 2018). The initial tier of such approaches is constructed to be protective of human health, often integrating high throughput assays (e.g., high throughput transcriptomics (HTTr) (Farmahin et al., 2017; Harrill et al., 2019) with more targeted tools (e.g., functional or binding assays for specific receptors) allowing broad biological coverage (Middleton et al., 2022; Zobl et al., 2024). Using multiple concentrations, points of departure (PoDs) can be calculated to identify concentrations at which a compound starts to cause biological perturbations (bioactivity) in a test system. These approaches have been evaluated in several case studies by calculating bioactivity:exposure ratios (BERs) from PoDs in combination with predicted systemic adult exposure estimates using physiologically based kinetic (PBK) models. Results from these evaluations demonstrate the protectiveness of these NAM based approaches mostly for systemic safety assessments (Baltazar et al., 2020; Dent et al., 2021; Middleton et al., 2022; Zobl et al., 2024; Cable et al., 2024). If needed an early tier can be followed up with more physiologically relevant cell systems for hazard testing or exposure predictions to refine outcomes (Thomas et al., 2019; Berggren et al., 2017). These new approaches have the potential to fundamentally transform chemical regulatory framework(s) by allowing more human-relevant decision-making to support sound human health safety decisions in diverse industrial sectors (cosmetics, industrial chemicals, pharmaceuticals, occupational health, etc.) (Magurany et al., 2023; Schmeisser et al., 2023).

To perform a comprehensive chemical safety assessment, it is crucial to ensure human exposures will not cause developmental and reproductive toxicity (DART). Due to the complexity and the distinct stages within the reproductive cycle, this was historically addressed using several OECD *in vivo* test guidelines (Knight et al., 2023) which assess changes in male and female reproductive function, gamete development and maturation, conception and embryo implantation, embryonic and fetal development, birth and weaning, the onset of puberty, attainment of full sexual function, and potential effects on subsequent generations (summarized in (EMA, 2023)). These DART-specific testing guidelines are employed to assess defined apical endpoints related to developmental or reproductive toxicity, such as pregnancy duration, fetal malformations, and the weight and morphology of reproductive organs, etc., but also evaluate non-specific/systemic effects like the body weight of the parental generation and the offspring, as well as the weight and morphological changes of reproductive as well as non-reproductive organs. The integration of DART and systemic testing endpoints serves as an approach protective of critical effect levels for human adverse outcomes (Browne et al., 2024). The first indication that NGRA approaches could also be protective for DART came from a study performed under the international government-to-government initiative “Accelerating the Pace of Chemical Risk Assessment (APCRA)”. By comparing PoDs from high-throughput assays with traditional hazard information for over 400 chemicals, including results from DART testing guidelines, this study demonstrated that for 89% of the compounds, the PoDs from NAMs were more conservative than PoDs derived from animal studies. No enrichment was found for compounds with data from DART studies within the cohort of 48 compounds in which the *in vivo* PoD was lower (Paul Friedman et al., 2020).

**FIGURE 1:**
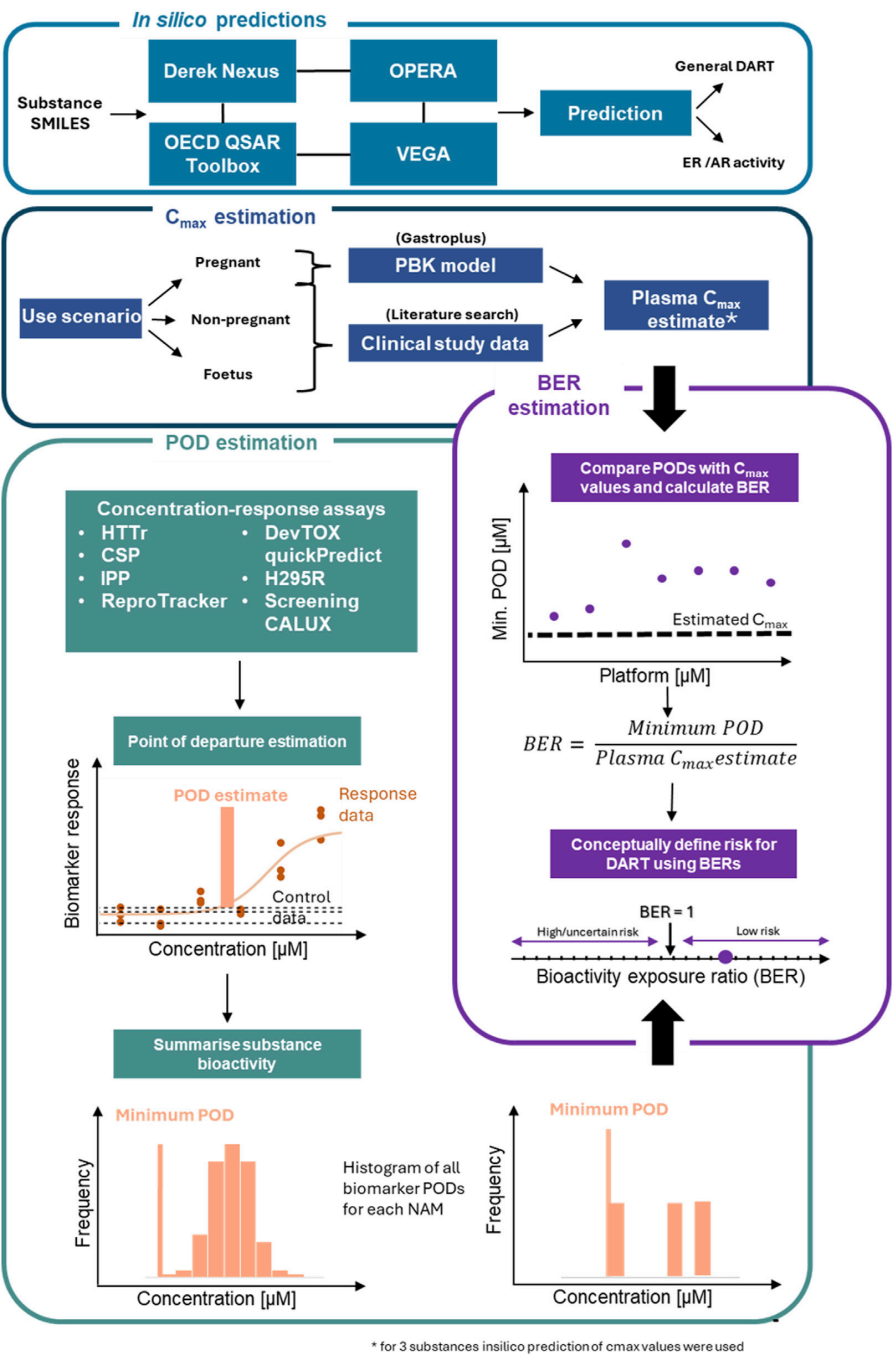
Workflow for evaluation of DART framework. The *in silico* module (tier 0 of the framework) includes 4 tools used to make *in silico* predictions on the benchmark chemicals for general DART toxicity and ER/AR activity. Tier one of the framework consists of 3 additional modules. The Cmax module includes a workflow to derive Cmax values for the chemical use-scenario for the 3 populations. In the PoD estimation module, 7 NAMs are processed to identify a minimum PoD. Outputs from Cmax estimation and PoD estimation modules are combined in the final module to estimate a BER for use in risk assessment. Schematic adapted from Middleton et al., 2022.

Previously we proposed an NGRA framework for DART (Rajagopal et al., 2022). The biological coverage of the NAMs within the proposed framework was evaluated by comparing cellular processes, signalling pathways and genes involved in known key stages in human reproduction and embryo-fetal development from an automated literature extraction to the read-outs from our NAM toolbox (including basic expression levels of cell lines). We showed ∼80% coverage of these processes based on gene numbers (Rajagopal et al., 2022). Knowledge of the biological coverage of our proposed framework and the previous work from APCRA (Paul Friedman et al., 2020) suggests that an NGRA approach could provide protection for DART, however conclusive evidence is still lacking. Therefore, in this study, we evaluated the protectiveness of our DART NGRA framework by testing 37 benchmark compounds. High and low-risk exposure scenarios for the 37 compounds were identified using DART-relevant data from authoritative sources as benchmarks. Within tier 0 of the framework *in silico* predictions covering general alerts for DART as well as for specific receptor activity were performed and results were compared to historical data to evaluate the predictive power of these tools. In tier 1 data from our DART NAM toolbox was generated and PoDs were calculated to estimate chemical bioactivity. Bioactivity was then compared to the estimated human exposure to calculate a BER for each exposure scenario (for an overview see Figure 1 and for a more detailed description of the workflow for the evaluation see material and methods).

To cover the different life stages of the reproductive cycle, it is essential to consider the exposure of non-pregnant adults, pregnant women, and fetal populations. This approach needs to take into account the anatomical and physiological changes in the pregnant woman and the gestational changes within the embryo, which may alter the absorption, distribution, metabolism, and excretion (ADME) of a compound, thereby impacting systemic exposure (Kapraun et al., 2019; Hudson et al., 2023). To broadly investigate the impact of population-based changes in pregnancy on internal exposures, clinical data from pregnant and fetal exposure as well as non-pregnant adult exposure was extracted from literature for the 37 compounds where available to inform on the exposure and exposure distribution between the three populations. PBK modelling was used to predict the internal exposure of the benchmark chemicals in female adults where no *in vivo* data could be found. BERs were calculated for all three subpopulations for each exposure scenario.

For the final evaluation of the NGRA approach BERs were used to group exposure scenarios into uncertain (BER <1) or low risk (BER >1). Conceptually a BER of 1 indicates that bioactivity would not be observed at human-relevant exposures. However, an experimentally derived BER threshold that would be considered protective for DART has not yet been proposed or agreed. Therefore, the purpose of this study was to assess whether a BER of 1 would be a protective of DART in humans and useful for decision making.

## 2 Materials and methods

### 2.1 Workflow for the evaluation of a DART NGRA framework

To evaluate the overall protectiveness of the DART framework, high and low-risk exposure scenarios for all compounds were identified where possible using DART-relevant data from authoritative sources as benchmarks. In the initial tier 0, *in silico* predictions using various tools, namely, Derek Nexus (Marchant et al., 2008), OECD QSAR Toolbox (https://qsartoolbox.org/), VEGA (Benfenati et al., 2013), OPERA (Mansouri et al., 2016) relevant to general DART hazard and estrogen and androgen activity were generated and compared to information from the same regulatory sources or, in the case of estrogen receptor (ER) and androgen receptor (AR), from the CompTox Chemicals Dashboard (see Figure 1). In tier 1 of the framework, data was generated for all NAMs in the toolbox to estimate chemical bioactivity across these NAMs, alongside an estimate of human chemical exposure via various methods (see Figure 1). The previously outlined conceptual design for our DART NGRA framework integrates broad, untargeted tools to detect bioactivity, including HTTr in three cell lines and a cell stress panel (CSP), with additional targeted NAMs—namely, *in vitro* pharmacological profiling (IPP) across 72 molecular targets and two human induced pluripotent stem cell (hiPSCs)-based assays (Rajagopal et al., 2022). These targeted NAMs provide more mechanistic based information and have been included to capture key molecular initiating events (MIEs) known to be important for DART, as well as fundamental cellular processes (e.g., iPSC metabolism and differentiation) that take place during early embryonic development. These assays provide complementary information to the broader untargeted assessments of bioactivity provided by the HTTr and CSP. Downstream events in the ER and AR pathways, along with disruptions in estrogen and androgen hormone synthesis, were identified as gaps in the previously published framework (Rajagopal et al., 2022). Since OECD-approved test systems for ER, AR and steroidogenesis (OECD, 2021; OECD, 2023b; OECD, 2023a) are commercially available, we incorporated these assays, namely, the H295R and AR and ER CALUX assays, to address these gaps during this evaluation. PoDs are calculated for each NAM and are compared to internal exposure to estimate a BER. By comparing the lowest PoD across all available NAMs to the estimated chemical exposure (plasma Cmax), a BER was calculated to characterize the risk of the compound at the specific exposure scenario with respect to DART. A conceptual BER of 1 was used as a threshold to differentiate between high/uncertain-risk (BER <1) and low-risk (BER >1) exposure scenarios, and protectiveness was evaluated by comparison to traditional risk assessment decisions. For simplification true dose estimates (Nicol et al., 2024), metabolism of a substance (Thomas et al., 2019) as well as uncertainty calculations (Middleton et al., 2022; Canada, 2021) to allow for decision-making, are excluded for this proof-of-concept study.

### 2.2 Benchmark chemical-exposure scenarios

In total 37 benchmark compounds were selected for evaluation of the DART NGRA framework. Compounds were selected to provide at least one human exposure scenario, and to include a variety of different consumer uses (e.g., pharmaceutical, cosmetic, plant protection, food), with routes of exposure including oral, dermal and intravenous administration. In total there are 49 chemical exposure scenarios across the 37 compounds (see Table 1).

**TABLE 1:** Exposure risk classifications for selected benchmark compounds.

Chemical	CAS number	Exposure scenario	Exposure risk
1,2-Octanediol	1117-86-8	Cosmetic, 5% in body lotion	Low Risk
2-Amino-6-chloro-4-nitrophenol	6358-09-4	Cosmetic, 2% in hair colourant	Low Risk
2-Ethylhexanoic acid (2-EHA)	149-57-5	Dietary, 3.1 mg/daily	Uncertain Risk
2-Methylresorcinol	608-25-3	Cosmetic, 1.8% in hair colourant	Low Risk
Aspartame	22839-47-0	Dietary, 2,400 mg/daily	Low Risk
all-trans-retinoic acid (ATRA)	302-79-4	Pharmaceutical, 0.1% cream	Uncertain Risk
all-trans-retinoic acid (ATRA)	302-79-4	Pharmaceutical, 80 mg/daily	High Risk
all-trans-retinoic acid (ATRA)	302-79-4	Dietary, <10,000 IU Retinol	Low Risk
Butylated hydroxytoluene (BHT)	128-37-0	Cosmetic, aggregate (max 0.8%)	Low Risk
2-Hydroxy-4-methoxybenzophenone, Oxybenzone, Benzophenone-3 (BP3)	131-57-7	Cosmetic, 6% in sunscreen	Low Risk
Caffeine	58-08-2	Dietary, 100 mg/daily	Low Risk
Caffeine	58-08-2	Dietary, 400 mg/daily	High Risk
Caffeine	58-08-2	Cosmetic, 2% in shampoo	Low Risk
Chlorpyrifos	2921-88-2	Dietary, 0.0045 mg/daily	Uncertain Risk
Chlorpyrifos	2921-88-2	Prenatal Exposure	High Risk
Cyclophosphamide	6055-19-2	Pharmaceutical, 60 mg/daily	High Risk
Cypermethrin	52315-07-8	Dietary, 0.3 mg/daily	Low Risk
Dibutyl phthalate (DBP)	84-74-2	Dietary, 0.6 mg/daily	Low Risk
DEET	134-62-3	Pharmaceutical, 15% in insect repellant	Low Risk
Diethyl phthalate (DEP)	84-66-2	Cosmetic, aggregate (max 10%)	Low Risk
Diethylstilbestrol (DES)	56-53-1	Pharmaceutical, 0.5 mg/daily	High Risk
Dexamethasone	50-02-2	Pharmaceutical, 0.75 mg/daily	High Risk
Digoxin	20830-75-5	Pharmaceutical, 0.024 mg/daily	Low Risk
Dolutegravir	1051375-16-6	Pharmaceutical, 50 mg/daily	High Risk
Ethylzingerone	569646-79-3	Cosmetic, aggregate (max 2%)	Low Risk
Fenazaquin	120928-09-8	Dietary, 3 mg/daily	Low Risk
Glutaraldehyde	111-30-8	Dietary, 9.6 mg/daily	Low Risk
Glutaraldehyde	111-30-8	Cosmetic, 0.1% in body lotion	Low Risk
HC Red 3	2871-01-4	Cosmetic, 3% in hair colourant	Low Risk
Metformin	657-24-9	Pharmaceutical, 2,000 mg/daily	Low Risk
Metoclopramide	364-62-5	Pharmaceutical, 60 mg/daily	High Risk
Metoclopramide	364-62-5	Pharmaceutical, 10 mg/daily	Uncertain Risk
Methotrexate (MTX)	59-05-2	Pharmaceutical, 10 mg/weekly	High Risk
Nitrofurantoin	67-20-9	Pharmaceutical, 200 mg/daily	High Risk
Panthenol	16485-10-2	Cosmetic, 5.3% in body lotion	Low Risk
Paraquat	4685-14-7	Dietary, 0.27 mg/daily	Low Risk
Retinol	68-26-8	Cosmetic, 0.05% in body lotion	Low Risk
Retinol	68-26-8	Dietary, <10,000 IU	Low Risk
Rosiglitazone	122320-73-4	Pharmaceutical, 4 mg/daily	High Risk
Cyclamate	139-05-9	Dietary, 420 mg/daily	Low Risk
Salicylate	69-72-7	Cosmetic, aggregate (max 3%)	Low Risk
Salicylate	69-72-7	Pharmaceutical, 162.5 mg/daily	Uncertain Risk
Salicylate	69-72-7	Pharmaceutical, 800–6,000 mg/daily	High Risk
Thalidomide	50-35-1	Pharmaceutical, 50 mg/daily	High Risk
Theophylline	58-55-9	Pharmaceutical, 800 mg/daily	High Risk
Theophylline	58-55-9	Dietary, 0.14 mg/daily	Low Risk
Valproic acid (VPA)	99-66-1	Pharmaceutical, 600 mg/daily	High Risk
Valproic acid (VPA)	99-66-1	Pharmaceutical, 3,600 mg/daily	High Risk
Warfarin	81-81-2	Pharmaceutical, 5 mg/daily	High Risk

#### 2.2.1 Assignment of risk classifications to benchmark chemical-exposure scenarios

To evaluate the DART NGRA framework each of the 49 chemical-exposure scenarios had a risk classification assigned with respect to human developmental and reproductive toxicity. These chemical-exposure DART risk classifications are considered the ‘truth’ and determine if the NGRA framework is sufficiently protective. Each of the 49 exposure scenarios was classified as either high, low, or uncertain risk for DART (Table 1). The risk classifications for each chemical-exposure scenario were determined based on the availability of existing toxicological information from animal studies and from evidence of developmental or reproductive effects in humans. In most cases, authoritative sources (e.g., EFSA, ECHA, EMA, FDA, EPA, SCCS risk assessments and/or reviews) were used to establish the risk classification for each chemical exposure scenario. Occasionally, other data sources were utilized to make risk classification decisions. For example, a biomonitoring study of prenatal exposure to chlorpyrifos was used to assign a risk classification for that specific exposure, based on human outcome and compound concentration in cord blood at birth. Additionally, literature searches were sometimes conducted to identify case reports that could provide evidence to support the assignment of either a high or low risk to human health. For 5 exposure scenarios it was not possible based on the available data to state with high confidence that an exposure was high or low risk, and therefore these scenarios were classified as uncertain. More detail on the 49 separate benchmark chemical-exposure scenarios, as well as the associated risk classifications and reasoning for these, including conclusions from regulatory opinions where available, can be found in Supplementary Data Sheet 1.

### 2.3 In silico predictions

There are numerous *in silico* tools available to predict general DART effects, as well as specific modes of actions (MoAs) such as estrogen (ER), androgen (AR), or thyroid (THR) binding and activation. For this work 14 models within four platforms Derek Nexus (Lhasa Limited)v. 6.2.0 (Marchant et al., 2008), OECD QSAR Toolbox (https://qsartoolbox.org/), VEGA (Benfenati et al., 2013) and Open (Quantitative) Structure-activity/property Relationship App (OPERA) v.2.8 (Mansouri et al., 2016) have been selected. The models are summarized in Supplementary Data Sheet 2, Table S1 and will not be discussed here as they have been described elsewhere (Weyrich et al., 2022). Briefly, the selected models represent Structural Alerts (SAs) and rule-based, expert knowledge or Quantitative Structure–Activity Relationship (QSAR) type of models. To identify the predictive potential of the selected models, an evaluation has been undertaken using data from DART relevant studies collated from the literature (see Supplementary Data Sheet 2). It is also worthwhile to mention that for the evaluation of Derek Nexus, two subsets of endpoints have been selected: the first one with 17 endpoints relevant to DART and the second with 34 endpoints relevant to both DART and systemic toxicity (see Table 2). Based on the predictive performance from the evaluation (see Supplementary Data Sheet 2, Tables S2-S4) as well based on the hands-on experience with the selected models, seven models have been chosen for the final battery of *in silico* models used in the DART Framework. These are Derek Nexus with selected endpoints, OECD QSAR Toolbox DART Scheme, VEGA_DEVTOX_PG, VEGA_ANDROGEN_COMPARA, VEGA_ESTROGEN_CERAPP, OPERA CERAPP and OPERA CoMPARA. The first three models are predicting general DART toxicity, and the last four are MoA specific models predicting binding affinity towards estrogen and androgen receptors. Additionally, OPERA models are also predicting agonist and antagonist activity. To simplify the interpretation of the prediction results, only binary outputs have been considered here without applicability domain or reliability/confidence information when available.

**TABLE 2:** Selected endpoints from Derek Nexus, with the first 17 endpoints defined as relevant to DART.

ID	Endpoint	ID	Endpoint
1	Bone marrow toxicity	18	Bladder urothelial hyperplasia
2	Cardiotoxicity	19	Bladder disorders
3	HERG channel inhibition *in vitro*	20	Cumulative effect on white cell count and immunology
4	Methaemoglobinaemia	21	Carcinogenicity
5	Oestrogenicity	22	Bradycardia
6	Peroxisome proliferation	23	Cyanide-type effects
7	Androgen receptor modulation	24	alpha-2-mu-Globulin nephropathy
8	Glucocorticoid receptor agonism	25	Nephrotoxicity
9	Oestrogen receptor modulation	26	Kidney disorders
10	5alpha-Reductase inhibition	27	Kidney function-related toxicity
11	Uncoupler of oxidative phosphorylation	28	Neurotoxicity
12	Mitochondrial dysfunction	29	Hepatotoxicity
13	Cholinesterase inhibition	30	Pulmonary toxicity
14	Thyroid toxicity	31	Ocular toxicity
15	Developmental toxicity	32	Splenotoxicity
16	Teratogenicity	33	Urolithiasis
17	Testicular toxicity	34	Adrenal gland toxicity

The chemical structures for the 37 selected benchmark substances have been obtained via the CompTox Chemicals Dashboard (https://comptox.epa.gov/dashboard/) as SMILES (Simplified Molecular Input Line Entry System). In the next step, the structures have been curated in terms of desalting and neutralising.

To evaluate the performance of the *in silico* models predicting general DART hazard (see above) we first needed to establish a source of truth to assess the predictions against. We decided to categorize each of the 37 chemicals as toxic or non-toxic. For a chemical to be categorized as toxic, there had to be evidence of developmental or reproductive toxicity in animal or human, irrespective of exposure/dose administration. The same data sources were used for this classification as for the chemical-exposure risk classifications, however for this exercise exposure was not considered, only presence or absence of effect. For four compounds (DEET, Nitrofurantoin, Cyclamate, and Aspartame), categorization was not possible due to uncertainty in the data. More detail on the assignment of a chemical as toxic or non-toxic can be found in Supplementary Data Sheet 1.

Similarly, for the models that predict MoA-based toxicity (e.g., Estrogen Receptor activation or Androgen Receptor activation), a source of truth was required to assess the predictions. For this purpose, we used the outputs from the ToxCast ER pathway AUC model (Judson et al., 2015) and the ToxCast AR pathway AUC model (Kleinstreuer et al., 2017), both are available via the CompTox Chemicals Dashboard. We were able to obtain this information for only 22 of the benchmark substances.

The predictive performance of *in silico* models has been described by following parameters: sensitivity measuring the ability to correctly predict positive (toxic) compounds

$$SE=\frac{\text{TP}}{\text{TP}+\text{FN}}$$

specificity measuring the ability to predict negative (non -toxic) compounds

$$SP=\frac{TN}{FP+TN}$$

accuracy assessing overall prediction performance by returning the fraction of compounds that were correctly predicted

$$ACC=\frac{\text{TP}+\text{TN}}{\text{TP}+\text{TN}+\text{FP}+\text{FN}}$$

balanced accuracy assessing overall model performance while giving each class equal weight

$$\text{BA}=\frac{\text{SE}+\text{SP}}{2}$$

and coverage assessing the proportion of compounds for which the model can make positive or negative prediction

$$\text{COV}=\frac{\text{TP}+\text{TN}+\text{FP}+\text{FN}}{\text{Total}}$$

using the variables: true positive (TP), false negative (FN), true negative (TN), and false positive (FP).

### 2.4 Computing chemical space

An in-house algorithm was developed to compute chemical space, where all chemicals (benchmark and evaluation) were represented by molecular descriptors computed using the python library RDKit [library version: 2023.03.2, python version: 3.11.14]. The dataset then underwent a first reduction stage through active removal of descriptors if the maximum tolerated cross-correlation (defined as Pearson’s r2) and minimum accepted diversity criteria were not met (0.8 and 0.3, respectively). A second reduction stage was then performed via PCA to the number of components needed to explain a desired amount of variance. The final reduction stage was carried out using the t-distributed Stochastic Neighbour Embedding (t-SNE) technique to project the dataset onto two dimensions and to visualise it graphically (Cable et al., 2024)]

### 2.5 Exposure and PBK modelling

**FIGURE 2:**
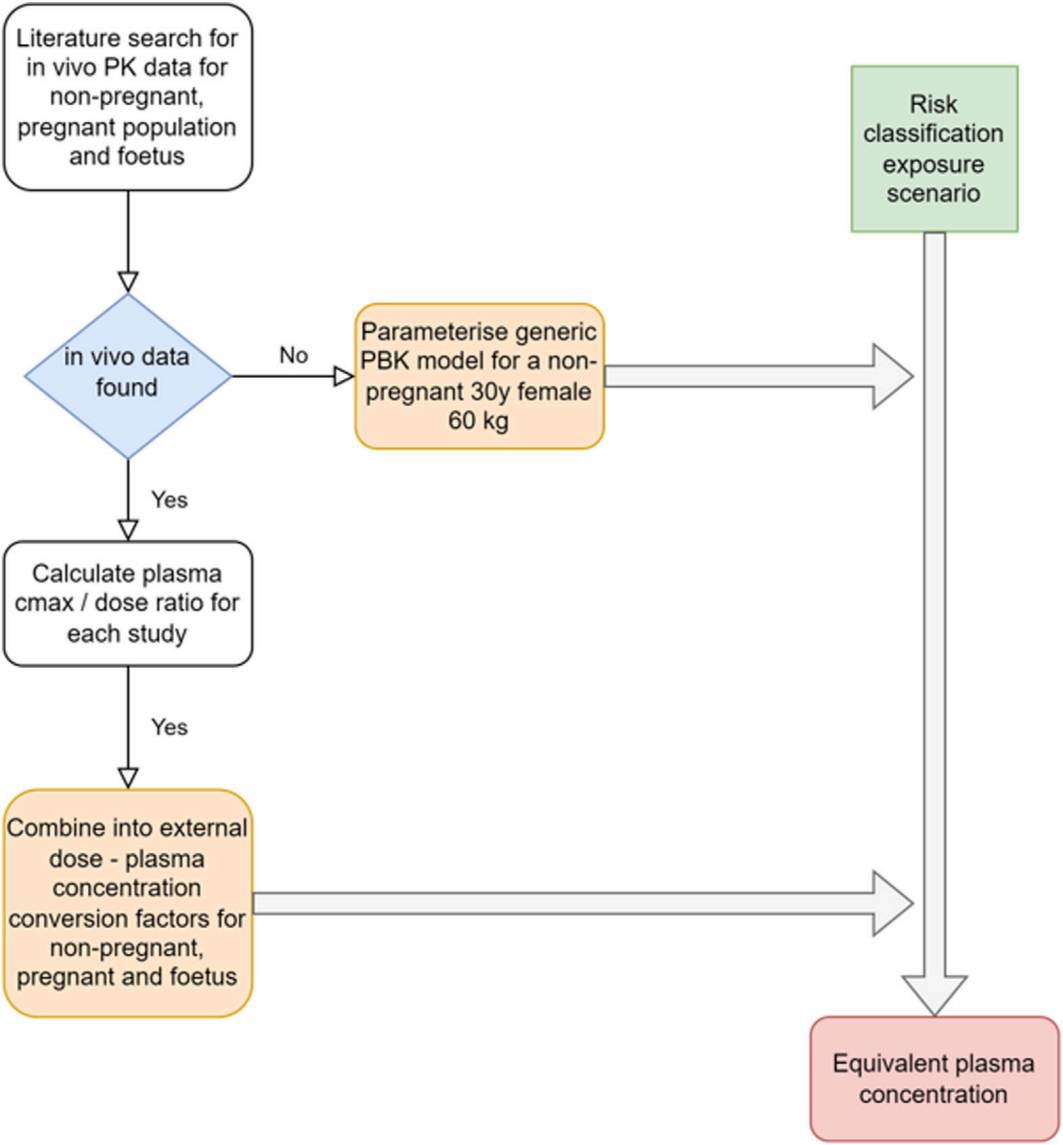
Schematic of the approach used to obtain systemic exposure values for the risk classification exposure scenarios.

The approach applied to obtain estimates of systemic exposures from *in vivo* PK data or through PBK modeling for the risk classification scenarios for the population groups of interest is illustrated in Figure 2.

#### 2.5.1 PK datamining for non-pregnant and pregnancy

For the benchmark chemicals (see Table 1), we systematically searched the literature in PubMed for pharmacokinetic (PK) studies and other systemic (plasma, serum, cord blood) concentration data in non-pregnant and pregnant populations. To collate the largest datasets, different combination of search keywords (see Table 3) was used. PK studies were categorized by the type of studies (see Table 4) depending on the dose and frequency of blood sampling information provided in those studies.

**TABLE 3:** Combination of primary and secondary search keywords used to identify relevant PK studies.

Criteria	Search keywords
Primary	*Caffeine* AND/OR *1-methyltheobromine* AND/OR *7-methyltheophylline*
	*Pharmacokinetics* AND/OR *absorption, distribution, metabolism and excretion* AND/OR *ADME* AND/OR *Relative Bioavailability* AND/OR *Bioequivalence* AND/OR *Toxicokinetic*
	AND/OR
	*Oral* AND/OR *Intravenous* AND/OR *Dermal*
	AND/OR
	*Area under the curve* AND/OR *maximum plasma concentrations* AND/OR *AUC* AND/OR *Cmax*
	AND/OR
	*Healthy Adult* AND/OR *Pregnancy* AND/OR or *Trimester* AND/OR *Partum* AND/OR *Gestational* AND/OR *Delivery* AND/OR *Mother* AND/OR *Maternal*
	AND/OR
	*Placenta* AND/OR *Prenatal* AND/OR *Preterm* AND/OR *Foetus* AND/OR *Foetal* AND/OR *Umbilical cord blood* AND/OR *Amniotic fluid*
	AND/OR
	*Ex vivo placental transfer* AND/OR *Foetal to maternal (FM) ratio*
Secondary	*Physiologically based pharmacokinetics* AND/OR *PBPK* AND/OR *PBK* AND/OR *PBTK*
	AND/OR
	*Mother-Foetus PBPK Models*
	AND/OR
	*Biomonitoring* AND/OR *Blood* AND/OR *plasma* AND/OR Serum *concentration*
Exclusions	*Preclinical AND/OR Rodent AND/OR Rat AND/OR Mice AND/OR Primate Pharmacokinetic studies*

**TABLE 4:** Type of data found in PK studies across the non-pregnant and pregnant population.

Data type	Dose characteristics	Exposure metric characteristics
Clinical PK study	Dose defined by amount, frequency, duration	Cmax, AUC, time course data
Sparse PK	Dose defined by amount, frequency, duration	Only few blood sampling time points, not cmax, e.g., at delivery
Therapeutic Drug Monitoring Data (TDM)	Dose defined by amount, frequency, duration, chronic exposure	Assumed steady state concentrations
Biomonitoring Data (BM)	Dose often not defined, e.g., aggregate from multiple sources, etc.,; chronic exposure	Assumed steady state concentrations
Case Studies, e.g., poisoning cases	Dose often not known	Single value, timepoint uncertain

#### 2.5.2 PK data analysis

The collated human *in vivo* PK data (see Supplementary Data Sheet 3) was visualized and analysed based on the reported mean values and standard deviations for systemic concentrations (plasma/serum/umbilical cord blood). Where this data was not available or reported in a different format, data gaps were filled as follows:

• Mean: calculated from range as (highest-lowest value)/2
• Standard Deviation (SD):
  ○ From the range (difference between the maximum and minimum) assuming a normal distribution, where about 99.7% of the data falls within three standard deviations from the mean, therefore SD≈range/6

  ○ Converting the standard error of the mean (SEM): SD=SEM×√N with N = population size

The units of the applied (external) and systemic (internal) dose data reported in the PK studies were harmonized. External doses were converted to mg per day based on an assumed body weight of 70 kg or a body surface area of 1.7 m^2^ (estimated body surface area of a 70 kg (thus – ‘average adult’) human https://www.chemeurope.com/en/encyclopedia/Body_surface_area.html#google_vignette) and the number of doses per day.

Internal exposure values (concentrations) were converted to μmol/L based on the molecular weight of the undissociated desalted chemical species.

To obtain an overview of the available PK data for each chemical the reported mean values for systemic exposures were plotted against the respective externally applied doses (see Supplementary Data Sheet 3).

For each datapoint (reported mean exposure value and standard deviation from a single study) the internal to external dose ratio (concentration-dose ratio, CDR) in units of μM/mg/day was calculated. The concentration-dose ratios from all obtained studies for each compound was then combined into overall weighted mean concentration-dose-ratios and weighted standard deviations for non-pregnant, pregnant and fetus sub-populations considering the different population sizes of the different studies by applying formulas below as depicted in Figure 4:

Weighted Mean: Each mean concentration value is multiplied by its respective population size to get the weighted sum. This sum is then divided by the total population size.

$${\bar{x}}_{total}=\frac{\sum {\bar{x}}_{i}\times N_{i}}{N_{total}}$$

Weighted Standard Deviation:

$${SD}_{total}=\sqrt{\frac{\sum \left(N_{i}-1\right)\times {SD}_{i}^{2}+\sum N_{i}\times {\left({\bar{x}}_{i}-{\bar{x}}_{total}\right)}^{2}}{N_{total}-1}}$$

where:

• ( $N_{i}$ ) is the population size for each reported mean exposure value,
• ( ${SD}_{i}$ ) is the standard deviation for reported mean exposure value,
• ( ${\bar{x}}_{i}$ ) is the reported mean exposure value for a study,
• ( ${\bar{x}}_{total}$ ) is the weighted mean of the entire dataset,
• ( $N_{total}$ ) is the total population size i.e., sum of all individual population sizes.

This formula accounts for the variance within each reported mean value and the variance between the reported means from different studies.

#### 2.5.3 Calculation of toxicokinetic variability factors (TKVF)

To quantitatively describe the toxicokinetic variability within the different population groups (non-pregnant, pregnant, fetus) the Toxicokinetic Variability Factor (TKVF) was calculated.

The TKVF is defined as the ratio between the internal dose metrics in a “sensitive” individual (e.g., 95th or 99th percentile, p95 or p99) in a population to that in a “typical” individual (e.g., median or mean) (WHO/IPCS, 2005; WHO/IPCS, 2014). Here, we calculated the TKVF_95_ for a 95th percentile individual.

For each population group TKVFs were calculated from the weighted mean concentration dose ratio and the 95th percentile of the distribution of concentration-dose values derived from the weighted mean and weighted standard deviation and a z-score of 1.64 for p95 as follows:

$$p_{95}={\bar{x}}_{total}+z\times {SD}_{total}$$

$${TKVF}_{95}=\frac{p_{95}}{{\bar{x}}_{total}}$$

#### 2.5.4 PBK modelling

PBK models were developed using GastroPlus^®^ 9.8 (Simulation Plus, Lancaster, California). Models were built for each chemical-exposure scenario and parameterized with a combination of *in silico* and *in vitro* derived values for logP (logarithm of octanol-water partition coefficient), pKa (logarithm of acid dissociation constant), water solubility, unbound fraction in plasma (fup), blood: plasma ratio (Rbp), hepatic intrinsic clearance (CLint), and intestinal absorption (Peff). In silico parameter estimates were sourced using ADMET Predictor (v.10) and *in vitro* data were sourced from the literature (see Supplementary Data Sheet 4). The kidney clearance rate was determined by the formula fup × GFR. Tissue-to-plasma partitioning coefficients (Kt:p) were calculated in GastroPlus using the Berezhkovskiy method (Berezhkovskiy, 2004) as a default with the exception of 2-EHA and Diethyl- and Dibutyl phathalate for which the Rodger and Rowland method was used (Rodgers and Rowland, 2006). It was assumed that chemical distribution into all tissues is perfusion limited.

Adult consumers (i.e., consumers of reproductive age) were represented by 60 kg adult female. This was selected as it was considered conservative both in terms of body weight, and potential use of cosmetics (SCCS Members, 2021).

Where a SED (systemic exposure dose) was reported systemic exposure from a dermal administration was modelled as a slow intravenous infusion of the SED, which corrects the applied dose for the rate of skin absorption (%). Where no SED was available dermal exposure route was predicted by the dermal module in GastroPlus^®^ administration.

The simulations were run until steady state was reached unless the described exposure scenario specified a specific duration of exposure.

### 2.6 Bioactivity measurements

#### 2.6.1 *In vitro* pharmacological profiling (IPP)

*In vitro* pharmacological profiling is used to measure specific and high affinity non-covalent binding interactions between chemicals of interest and various targets with known safety liabilities. These targets include G protein coupled receptors (GPCRs), nuclear hormone receptors (NHRs), ion channels and enzymes. For this evaluation we included 72 targets of interest a full list of which is available in Supplementary Data Sheet 5, across a range of radioligand binding, enzymatic and protein-protein interaction assays run in binding mode only. Forty-four of the targets in the IPP panel have been associated with *in vivo* adverse drug reactions by the pharmaceutical industry (Bowes et al., 2012; Brennan et al., 2024) and these were supplemented with an additional 28 targets to expand coverage of DART relevant targets based on a literature search (Rajagopal et al., 2022; Wu et al., 2013) or for their finding as targets from cosmetics (Burbank et al., 2024).

The process for deriving a PoD for each of the 72 targets consisted of a two-step method that has been widely adopted when conducting *in vitro* pharmacological profiling experiments (Brennan et al., 2024). The first step consists of a screening phase whereby compounds are screened at a single concentration in two replicates (either 10 or 100 μM depending on solubility and cytotoxicity information). Targets showing an inhibition or stimulation greater than 50% of a maximal response produced by a reference compound are followed up in a second phase which includes an eight-point concentration response (in two replicates). The choice of concentrations was informed by the % of inhibition/stimulation from the screening phase so that both plateaus in the sigmoid curves are sampled. EC50 values (concentration producing a half-maximal response) and IC50 values (concentration causing a half-maximal inhibition of the control agonist response) were determined by the Bayesian probabilistic model of the concentration-response curves and the Hill equation as per (Labelle et al., 2019). The priors for IC50 were set to the median experimental dose, the slope was set to 1.0 and low and high dose responses were set to 0% and 100%, respectively. Calculated IC50s were taken forwards as the IPP PoDs (Middleton et al., 2022).

IPP target was split into DART targets as well as broad screening targets by using information received from the original publications (Bowes et al., 2012; Brennan et al., 2024), the AOP wiki (https://aopwiki.org/) the Integrated Chemical Environment (ICE) database (ICE: Integrated Chemical Environment (nih.gov)) and mouse genome informatics database (https://www.informatics.jax.org/). Using these data sources, from the 72 targets within the IPP panel 49 could be identified as DART relevant (see Supplementary Data Sheet 5).

#### 2.6.2 U2-OS ERα and AR CALUX^®^ pre-screens

Perturbing the ER or AR pathways can cause endocrine disruption, which may lead to DART. Upon compound binding to the ER or AR, the receptor is activated, entering the nucleus to bind to recognition sequences in promoter regions of target genes called hormone response elements (HRE). CALUX bioassays comprise human bone cell lines (U2-OS), incorporating the firefly luciferase reporter gene coupled to HRE, to identify compounds capable of activating the specific pathways linked to these response elements. By addition of the appropriate substrate for luciferase, light is emitted. The amount of light produced is proportional to the amount of ligand-specific pathway activation (or pathway inactivation, in the case of an antagonistic response), which is benchmarked against relevant reference compounds (Sonneveld et al., 2005).

To detect any direct ER or AR activity for the 37 compounds both the ERα CALUX and AR CALUX assays were performed as pre-screens as described in the OECD test guidelines 455 (OECD, 2021) and 458 (OECD, 2023b) respectively. The OECD test guidelines require a “comprehensive run”, in triplicate, to be conducted following a pre-screen. However, the pre-screens alone, also provide useful information on if a compound is an agonist or antagonist of the ER or AR and enable calculation of a lowest observed effect concentration (LOEC) for that activity. We have taken the pre-screen LOEC as the PoD for these two assays in our framework.

In addition to providing information on ER and AR activity for the 37 compounds, the data generated in the CALUX pre-screens were also used for interpretation of the H295R Steroidogenesis Assay, described in the next section.

#### 2.6.3 H295R steroidogenesis assay with AR/ER CALUX detection method

Steroidogenesis is the process by which steroid hormones (including estrogens and androgens) are synthesized mainly in the gonads and adrenal glands by a combination of pathways. Disruption of this process is a form of endocrine disruption which is a key mode of action leading to DART. The H295R assay uses a human adrenocarcinoma cell line which has the unique property of expressing all of the genes required for conversion of cholesterol to sex hormones (Haggard et al., 2018).

The H295R assay was performed as described in the OECD TG 456 (OECD, 2023a), with the exception that only one biological repeat was performed in this evaluation rather than the recommended two within the guideline. To quantify the levels of estrogens and androgens produced by the H295R cells after compound exposure, the assay medium was analysed on the ERα and AR CALUX^®^ bioassays. Briefly U2-OS cells were treated with diluted H295R supernatant, and the hormone levels present within the supernatant were quantified as reporter gene activities. Changes in hormone levels compared to a vehicle control (DMSO) exposure indicate that certain enzymes involved in steroidogenesis were being affected by the test compound. In order to rule out carry over of ERα or AR active compounds in the H295R media, and to ensure that reporter gene activity was only due to changes in hormone levels in the H295R cells, the data from the CALUX pre-screen was used to rule out carry over of ER or AR active compounds in the media. For detailed methods on this approach see Nikopaschou et al., 2023; Nikopaschou et al., 2023).

#### 2.6.4 ReproTracker

The ReproTracker assay assesses chemical perturbation of early embryonic development by evaluating key events of cardiomyocyte, hepatocyte-like (HLC) and neuronal cell differentiation using human induced pluripotent stem cells (hiPSCs). The ReproTracker protocol is described in (Jamalpoor et al., 2022; Moreau et al., 2023) and was followed introducing a few modifications to allow for concentration response analysis. Briefly, following initial dose range finding experiment in undifferentiated hiPSC, six non-cytotoxic concentrations of test substance (1:3 dilution) and solvent controls were tested in tri-lineage differentiation experiments in biological triplicate. Lineage specific differentiation was then investigated by assessment of gene expression patterns of cell-specific biomarkers, induced cytotoxicity (AlamarBlue cell viability assay) and by morphological profiling. Gene biomarkers quantified by multiplex qRT-PCR included *BMP4* and *MYH6* for cardiomyocytes, *FOXA2* and *AFP* for hepatocyte-like cells, *PAX6* and *NESTIN* for neural rosette lineage. Dose range of thalidomide was included in each cardiomyocyte and HLC differentiation experiment as a positive control, whereas retinoic acid served as a positive control in neural rosette differentiation. Saccharin was used as a negative control substance for all 3 lineages.

For dose dependent qRT-PCR analysis quality control filtered Ct values were normalised using an adaptation of the Pfaffl (Pfaffl, 2001) method to calculate ΔCt values with respect to biomarker and housekeeping gene amplification efficiencies. BMDExpress2 (Phillips et al., 2019) dose response modelling methods have been applied independently for all lineages and timepoints where Williams Trend Test filter was applied (p < 0.05 and fold change≥1.5) and 6 models (Poly 2, Hill, Power, Exponential 3, 4 and 5, with recommended default configurations) were fit. Benchmark concentration/dose (BMD) and lower (BMDL) values were calculated for each concentration response, based on a benchmark response (BMR) factor of 10% using the model which produced the lowest Akaike Information Criterion (AIC) value. Concentration responses and estimated BMDs were deemed as significant when the BMD for the response was under the highest concentration tested and when the BMD upper to lower ratio value (BMDU/BMDL ratio) was between 1.1 and 5,000 (filter set to remove under extrapolated values). Only BMDLs of a downregulated responses from 6 indicative biomarkers of developmental toxicity (BMP4 D7, MYH6 D14, FOXA2 D7, AFP D21, PAX6 D7 and 13 and NESTIN D13 (Jamalpoor et al., 2022) were considered significant PODs.

In addition, Alamar blue read outs were taken from the differentiating cells at day 7 and the end of each differentiation to measure cell viability in a dose dependent way. The AlamarBlue readout was normalised and transformed using a Bayesian hierarchical approach The Bayesian model assumes measurement between rows on the treatment plates are correlated but allows for differences in average sample response between rows. Such models reduce the plate effect between samples of different rows to increase confidence effects seen in a concentration response are due to a chemical effect A POD was calculated from sampled concentration response curves, from the posterior distribution of the model, where a 5% decrease from the baseline response is seen. Concentration dependency scores (CDS) represent the possible values of the POD distribution being below the highest tested concentration and therefore used as measure of statistical confidence that a response has been observed for the treatment, where a CDS over 0.5 was considered a confident hit. The final cell viability PODs were those at time point end (day 14 for cardiomyocytes, day 21 for HCLs and day 13 for neural) and had CDS above or equal to 0.5. Both the gene biomarker PODs and cell viability/cytotoxicity PODs for each tested lineage are considered for BER calculation.

#### 2.6.5 devTOX quickPredict

devTOX quickPredict is a human induced pluripotent stem cell (hiPSC) -based assay that predicts the concentration at which a compound may elicit developmental toxicity. The assay uses the metabolic perturbation of two biomarkers, ornithine and cystine, in a ratio (o/c ratio) to predict the concentration at which a test article shows developmental toxicity potential (dTP). Assays were performed as described in Palmer et al. (2013) with modifications. Briefly undifferentiated hiPSCs are exposed to the compound for 48 h, with media and test article replacement every 24 h. Ornithine and cysteine concentrations were measured from the final 24-h treatment using Ultra-Performance Liquid Chromatography-High Resolution Mass Spectrometry (UPLC-HRMS). Cell viability was assessed after sample collection using the CellTiter-Fluor Cell Viability Assay (Promega).

Dose-response analysis for the o/c ratio, cell viability, ornithine response and cystine response were performed with GraphPad Prism (version 9.1 or newer, GraphPad Software). Each data set was fit with a nonlinear model. The standard model used for analysis is a four-parameter log-logistic nonlinear model. However, the Akaike information criterion (GraphPad Prism) was used to determine if an asymmetric (five-parameter) or multiphasic nonlinear model was a better fit for the data than the four-parameter model. The developmental toxicity potential (dTP, o/c ratio) and toxicity potential (TP, cell viability) concentrations were predicted from the respective dose-response curves using the hiPSCs cell developmental toxicity threshold (dTT, 0.85).

#### 2.6.6 Cell stress panel (CSP)

The CSP used in our framework detects multiple mechanisms leading to cellular stress, including mitochondrial toxicity, DNA damage, inflammation, etc. Cell stress is a fundamental factor in many systemic and DART relevant adverse outcome pathways (AOPs), either as a molecular initiating event or as key event. It has also been reported as a key characteristic of male and female reproductive toxicants (Arzuaga et al., 2019; Luderer et al., 2019). Compounds were tested using the previously developed cell stress panel (Hatherell et al., 2020) and the expanded biomarker panel outlined in Middleton et al. (2022). HepG2 cells were treated with compounds for 24 h across 8 concentrations prior to biomarker analysis. The same plate layout and number of replicates (3 biological and 2 technical) were used as described previously for each assay within the panel.

#### 2.6.7 High throughput transcriptomics (HTTr)

HTTr measures transcriptional changes of biological perturbations caused by any interaction of a chemical with the cell. HTTr is a well-established method for determining bioactivity, chemical potency and mode of action across diverse chemistry. HepG2, HepaRG and MCF7 cells were treated with each compound for 24 h across a dose range of 7 concentrations and lysed using TempO-Seq lysis buffer (BioSpyder Technologies, proprietary kit, see Middleton et al. (2022) for method details). Sequencing was performed using TempO-Seq (BioClavis) version 2 of the human whole transcriptome panel and analysed as described previously in Middleton et al. (2022).

### 2.7 BER calculation

For each of the 49 exposure scenarios across the 37 chemicals, the ratio between a minimum platform PoD and the estimated Cmax is calculated giving the bioactivity exposure ratio. A minimum platform PoD is defined as the lowest PoD of the following possible platform PoDs or a subset of:

1. The minimum Bayesian derived PoD from the *in vitro* pharmacological profiling platform
2. The global PoD from the cell stress panel when analysed using the BIFROST method.
3. The global PoD from the HTTr platform (for each cell line tested) was derived using the BIFROST method.
4. The minimum BMDL from the HTTr platform (for each cell line tested) using BMDExpress2.
5. The minimum PoD from the ReproTracker cytotoxicity or gene biomarker dose response (for each lineage tested)
6. The minimum PoD from the devTOX quickPredict cytotoxicity or developmental toxicity potential (dTP) dose response.
7. The minimum LOEC from the H295R steroidogenesis assay
8. The minimum LOEC from the screening CALUX assay

### 2.8 Protectiveness and utility metrics

The protectiveness and utility metrics as defined in Middleton et al. were used to assess the overall performance of the toolbox and workflow. Using a BER threshold of 1, where exposure scenarios with BER <1 are determined as uncertain risk (i.e., not low risk) and those with BER >1 as low risk, we define protection as the percentage of high-risk exposure scenarios which are correctly identified as uncertain risk and utility gives the percentage of low-risk exposure scenarios which are correctly identified low-risk using.

## 3 Results

### 3.1 Chemical space and *in silico* predictions

**FIGURE 3:**
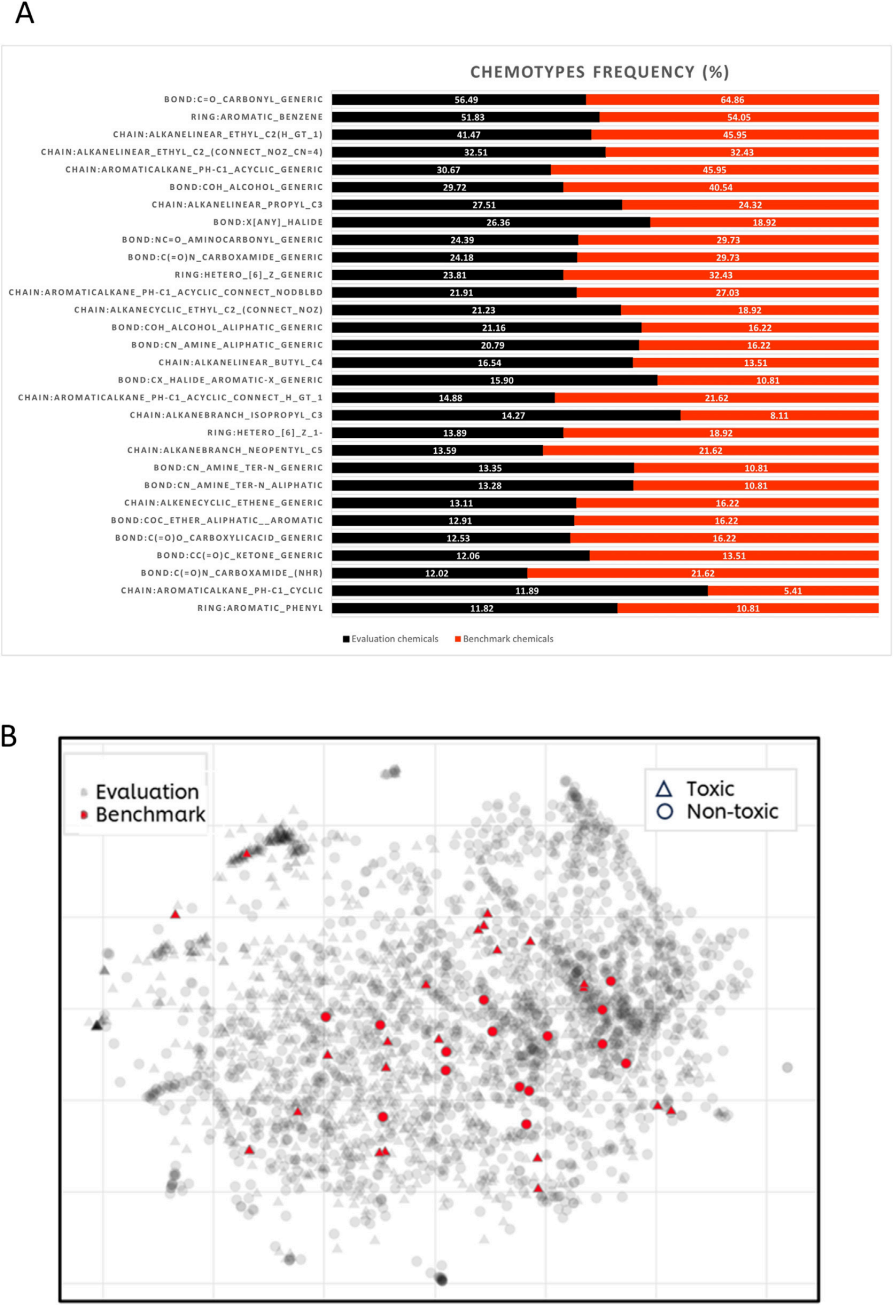
Structural diversity of benchmark and *in silico* evaluation chemicals. **(A)** Histogram of the 30 most frequent chemotypes present within benchmark (shown in red) and evaluation chemicals (shown in black). **(B)** t-SNE visualisation of the chemical space covered by benchmark (grey dots) and evaluation chemicals (red dots). The hazard categorisation (toxic/non-toxic) is displayed by the shapes, circle represents non-toxic and triangle- toxic substances.

To determine if the selected benchmark compounds fall within the same chemical applicability domain as the approximately 3,000 chemicals used for evaluating the *in silico* tools (see Supplementary Data Sheet 2), their chemical space and structural diversity were compared to those of the 37 benchmark chemicals (see Figure 3). An initial visual inspection of the 37 structures shows that majority of the chemicals are cyclic chemicals, with aromatics rings being the most frequent. Only a few chemicals represent the aliphatic, acyclic chemistry. Another remark is that most of the compounds have a carbonyl group. These observations have been confirmed by characterisation of the chemotypes with ToxPrint (Yang et al., 2015). From the 174 chemotypes identified in the 37 compounds, the two most frequent chemotypes are: bond:C=O_carbonyl_generic (present in 24 compounds) and ring:aromatic_benzene (present in 20 compounds) (see Figure 3A).

To investigate the structural diversity of the 37 chemicals, they were compared against the larger set of chemicals used for the initial evaluation of *in silico* models (see Supplementary Data Sheet 2). From the entire repository of 729 chemotypes, 513 chemotypes have been identified in the evaluation set. Figure 3A shows the first two most frequent chemotypes in both sets are: ring:aromatic_benzene and bond:C=O_carbonyl_generic. It can be also noticed that benchmark chemicals are also heavily represented by chemotypes describing presence of alcohols (bond:COH_alcohol_generic) and alkanes attached with aromatic rings (chain:aromaticAlkane_Ph-C1_acyclic_generic), both chemotypes with frequency above 40%. The structural diversity was also represented by the visualisation of chemical space of both datasets. As shown in Figure 3B, benchmark chemicals are scattered over most of the chemical space represented by the evaluation chemicals. There are also some regions within the evaluation chemical set which are not represented by benchmark chemicals. This is not surprising considering the small number of benchmark compounds comparing to the significantly larger evaluation set (37 vs. 2,944 compounds). Figure 3B illustrates that there are no structural differences between DART toxicants and non-toxicants, as both groups are evenly distributed.

### 3.2 Evaluation of tier 0 *in silico* predictions for DART

**FIGURE 4:**
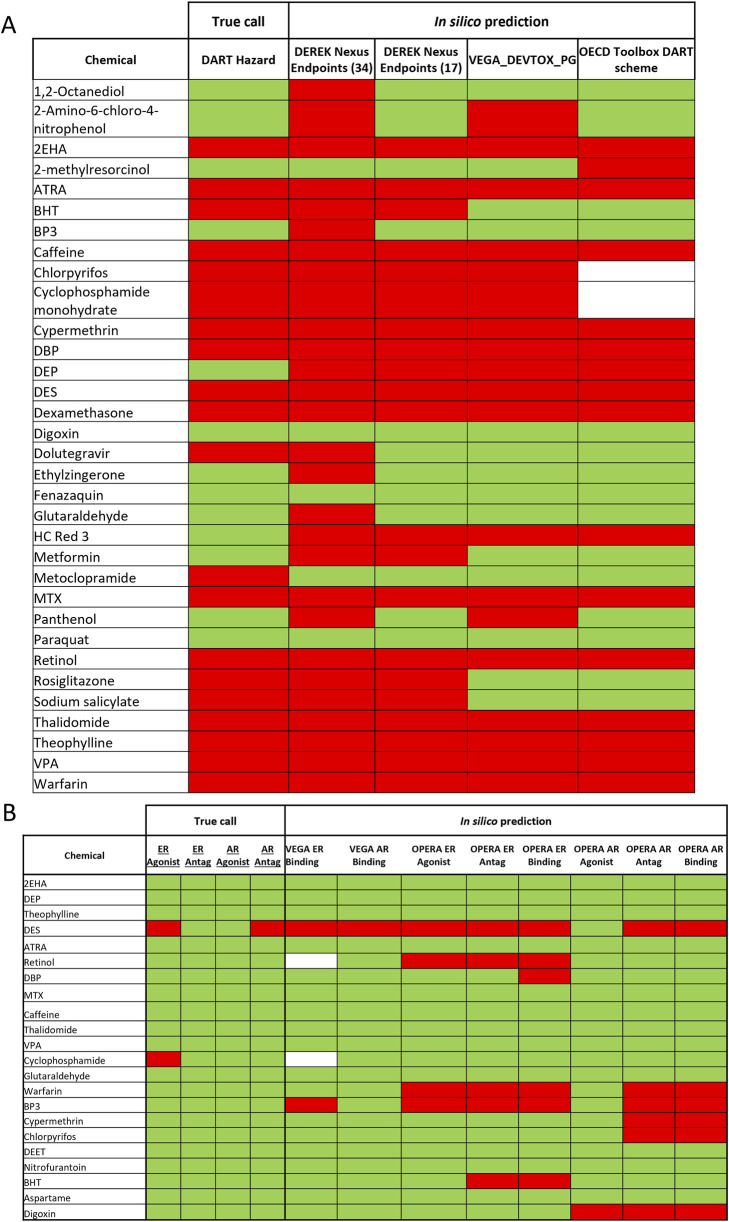
In silico predictions for the 37 benchmark chemicals. Results of the different *in silico* tools for prediction of general DART toxicity **(A)** and ER and AR activity **(B)** is shown in comparison to the “true call” hazard characterisation for each chemical. Green is indicating non-toxic/non-active and red indicating toxic/active and white–not predicted by the tool.

Most of the DART toxic benchmark chemicals (see Table 1) have been correctly identified by at least one of the general DART *in silico* models (see Figure 4A). Only one toxicant–Metoclopramide has been falsely predicted as non- toxicant by all models. Figure 4A and Table 5 show that Derek Nexus with 34 endpoints has the highest sensitivity (95%), with one false negative prediction. Reducing the Derek Nexus endpoints to the 17 endpoints identified as most relevant for DART (see Table 2) resulted in one more false negative chemical–Dolutegravir. The other two tools based on the P&G decision tree (OECD QSAR Toolbox DART Scheme and VEGA_DEVTOX_PG) have slightly lower sensitivity, each model incorrectly predicting the same five chemicals (BHT, Dolutegravir, Metoclopramide, Rosiglitazone and Sodium salicylate) as non-toxicant. Additionally, the DART Scheme in the OECD QSAR Toolbox was not able to categorise two toxic compounds (Chlorpyrifos and Cyclophosphamide) as the applicability domain of the tool is not covering organophosphorus compounds. From 13 non-toxicants, only three chemicals (Digoxin, Fenazaquin and Paraquat) have been correctly predicted by all models. Derek Nexus (34 endpoints) has produced the highest number of false positive predictions; nine compounds were predicted incorrectly giving very low specificity of ∼30%. This is not surprising as the broader set of endpoints covers both DART as well as systemic adverse effects. The other models predicted approximately 70% of non- toxicants correctly. Two DART non-toxicants (DEP and HC Red 3) were predicted as toxicants by all models. Overall, Derek Nexus with 17 selected endpoints provides the best performance in terms of accuracy (∼85%) with well-balanced sensitivity (90%) and specificity (77%). The two models based on the P&G decision tree gave similar predictive performance with accuracy above 70%. The Derek Nexus with 34 endpoints has the lowest accuracy caused by generating the highest number of false positive predictions.

**TABLE 5:** The predictive performance of *in silico* models for general DART toxicity.

20 tox and 13 non-tox	TP	FN	TN	FP	SE (%)	SPE (%)	ACC (%)	BA (%)	COV (%)
Derek Nexus (34 endpoint)	19	1	4	9	95.00	30.77	69.70	62.88	100.00
Derek Nexus (17 endpoints)	18	2	10	3	90.00	76.92	84.85	83.46	100.00
OECD Toolbox DART scheme	13	5	10	3	72.22	76.92	74.19	74.57	93.94
VEGA DevTox	15	5	9	4	75.00	69.23	72.73	72.12	100.00

TP, true positive, FN- false negative, TN, true negative; FP, false positive, SE- sensitivity = *TP*/(*TP* + *FN*), SP, specificity = TN/(TN + FP), ACC, Accuracy = (TP + TN)/(*TP* + *TN* + *FP* + *FN*), BA, Balanced Accuracy = (SE + SP)/2, COV, Coverage = (TP + TN + FP + FN)/Total.

Figure 4B compares the *in silico* predictions from four MoA specific models with the ER and AR active/inactive categorization for 22 compounds. DES was correctly predicted by all ER models, being identified as an ER agonist as well as antagonist in OPERA. All models do not predict any ER activity for cyclophosphamide monohydrate. DES, the only compound with known AR activity in the compounds set, is also correctly identified by all AR related models. Although all MoA specific models have been developed using the same ToxCast data, differences in the predictions can be observed (see Figure 3B). In general, the OPERA models predict more receptor binding/activity than corresponding models in the VEGA platform. Because of the small amount of ED active chemicals (only two actives from 22 compounds), evaluation with a different set of compounds would be needed to better reflect the predictive power of MoA specific models.

### 3.3 Exposure and PBK modelling

**FIGURE 5:**
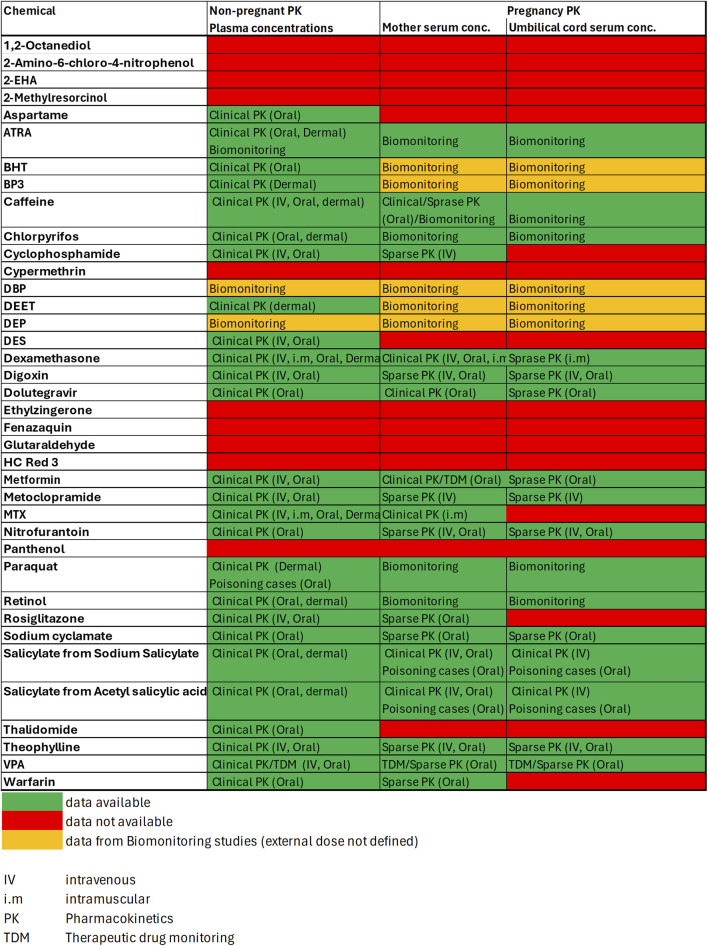
*In vivo* PK data availability matrix. Data is classified according to Table 4 into Clinical PK, Sparse PK, Therapeutic Drug Monitoring, Biomonitoring or Case Study data.

In a first step *in vivo* literature data were derived to obtain insights into observed internal concentrations for non-pregnant, pregnant and fetus sub-populations. Figure 5 shows, that for 23 out of the 37 chemicals some *in vivo* data was available which informed the internal exposure estimates. However, only for 12 of these 23 compounds data on systemic concentrations were available for both, mother and fetus (i.e., from serum and cord blood samples taken at birth). Where no *in vivo* data could be found, internal exposures were predicted using a generic PBK modelling for a non-pregnant population. From both, *in vivo* and PBK predicted plasma concentrations, concentration-dose ratio’s (CDR) were calculated and applied for the calculation of plasma concentrations for the risk classification scenarios of interest. A full summary of all concentrations-dose ratios can be found in the Supplementary Material (Supplementary Data Sheet 6).

### 3.4 Comparing intra- and inter-population exposure differences

**FIGURE 6:**
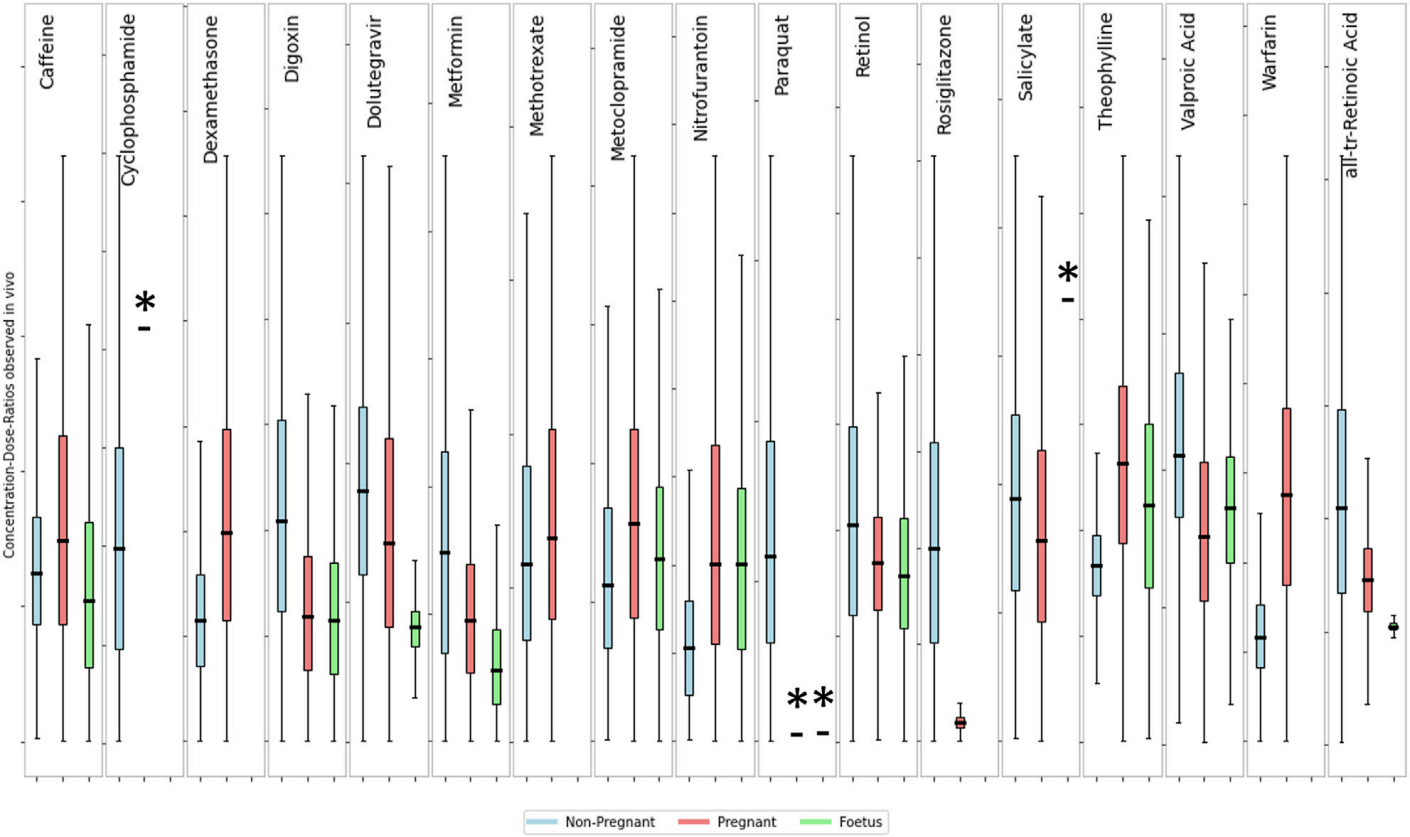
Comparison of concentration-dose ratios between non-pregnant, pregnant and foetus. The box and whisker plots show the fold-difference from the median of the distribution of concentrations-dose ratios for a non-pregnant (blue), pregnant (yellow) and foetal (green) sub-population. Boxes show the interquartile range with the First (Lower) Quartile being the midpoint of the lower half and the Third (Upper) Quartile the midpoint of the upper half of the data. Lower whiskers represent the lower boundary as the first quartile minus 1.5 times the interquartile range, upper whiskers show the upper boundary as the third quartile plus 1.5 times the interquartile range. The y-axis scales are not shown but are different between subplots. Actual values for the dose-concentration ratios are summarized in Table 5. * Indicates that data is from fewer than 10 subjects.

An analysis of the variability among the three subpopulations for the 12 substances with available data (see Figure 6) indicates that there is no clear separation between life stages. Showing that the variability within each life stage is greater than the difference between the means of those life stages. To quantitatively describe the population variability observed in the collated data, a toxicokinetic variability factor was calculated for each chemical and population group (see Table 6). This factor reflects the variability of pharmacokinetics within the population as well as any resulting effects of external factors such as different routes of exposure and formulations, etc. The fold differences between the mean concentration-dose-ratios of pregnant or fetus populations groups and a non-pregnant population was calculated to quantify the inter-population variability. Intra- and interpopulation variability are compared in Table 6.

**TABLE 6:** Intra- population variability and differences between non-pregnant, pregnant and fetus population groups.

Chemical	Population	Total population size	TKVF	CDR fold difference to non-pregnant	p-value	CDR fold difference to pregnant	p-value
VPA	Non-Pregnant	673	1.62	1.00			
VPA	Pregnant	112	1.85	**0.69**	0.00	1.00	
VPA	Fetus	33	1.52	**0.78**	0.00	1.13	0.11
Theophylline	Non-Pregnant	119	1.43	1.00			
Theophylline	Pregnant	62	1.73	**1.58**	0.00	1.00	
Theophylline	Fetus	26	1.81	1.33	0.11	0.84	0.11
Salicylate	Non-Pregnant	57	1.96	1.00			
Salicylate	Pregnant	16	2.34	0.79	0.26		
Salicylate	Fetus	3	1.95	*1.95*			
ATRA	Non-Pregnant	333	2.14	1.00			
ATRA	Pregnant	180	1.47	**0.77**	0.00	1.00	
ATRA	Fetus	10	1.06	**0.55**	0.00	**0.71**	0.00
Retinol	Non-Pregnant	56	2.32	1.00			
Retinol	Pregnant	180	1.64	0.88	0.30		
Retinol	Fetus	10	1.86	0.82	0.32		
Caffeine	Non-Pregnant	331	1.89	1.00			
Caffeine	Pregnant	264	2.42	**1.13**	0.05	1.00	
Caffeine	Fetus	1,687	2.84	**0.67**	0.00	**0.59**	0.00
Dolutegravir	Non-Pregnant	278	1.87	1.00			
Dolutegravir	Pregnant	101	2.64	**0.70**	0.00	1.00	
Dolutegravir	Fetus	22	1.40	**0.46**	0.00	**0.65**	0.00
Paraquat	Non-Pregnant	67	3.16	1.00			
Paraquat	Pregnant	4		0.05		1.00	
Paraquat	Fetus	3		0.06		1.15	
Digoxin	Non-Pregnant	211	2.32	1.00			
Digoxin	Pregnant	106	2.35	**0.55**	0.00	1.00	
Digoxin	Fetus	68	2.38	**0.54**	0.00	0.98	0.89
Nitrofurantoin	Non-Pregnant	281	3.36	1.00			
Nitrofurantoin	Pregnant	125	5.73	1.29	0.40	1.00	
Nitrofurantoin	Fetus	15	2.90	2.25	0.09	1.74	0.22
Metoclopramide	Non-Pregnant	37	2.85	1.00			
Metoclopramide	Pregnant	20	2.71	1.45	0.25	1.00	
Metoclopramide	Fetus	20	1.97	1.50	0.07	1.04	0.89
Metformin	Non-Pregnant	219	3.15	1.00			
Metformin	Pregnant	191	2.75	**0.73**	0.01	1.00	
Metformin	Fetus	144	2.99	**0.41**	0.00	**0.56**	0.00
MTX	Non-Pregnant	242	2.74	1.00			
MTX	Pregnant	20	2.65	1.11	0.68		
Cyclophosphamide	Non-Pregnant	154	3.15	1.00			
Cyclophosphamide	Pregnant	1		2.85			
Warfarin	Non-Pregnant	124	1.82	1.00			
Warfarin	Pregnant	21	1.93	**2.35**	0.00		
Rosiglitazone	Non-Pregnant	233	4.34	1.00			
Rosiglitazone	Pregnant	31	1.71	**0.19**	0.00		
Dexamethasone	Non-Pregnant	99	1.99	1.00			
Dexamethasone	Pregnant	83	2.51	**1.50**	0.00		

Marked in green are values that are significantly lower, in red those which are higher compared to the CDR, for Non-Pregnant. In cursive grey are values for which the population size was considered too small (<10) to calculate any statistics (standard deviation, TKVF, and p-value).

The toxicokinetic intra-population variability as characterized by the TKVF ranged from 1.06 to 5.73, with a mean of 2.36. The fold difference between pregnant/fetus and non-pregnant concentration-dose ratios was in the range 0.19–2.35, i.e., for most of the chemicals the variability within a population group was greater than the differences observed between populations suggesting that in most cases variability caused by pregnancy or due to gestational changes is within the toxicokinetic variability in the general population. More importantly, for the majority of chemicals the fold difference between pregnant/fetus and non-pregnant was less than one, meaning that the internal exposure resulting from the same external exposure was lower in the pregnant/fetus population group. Exceptions are Caffeine, Theophylline, Warfarin and Dexamethasone for which the fold differences between means were 1.13, 1.58, 2.35 and 1.50, respectively. Overall, the analysis of the data shows that in most cases internal exposure estimates for a general population–considering variability within the population - would cover the exposures in the pregnant and fetal sub-group.

### 3.5 Is tier one of the DART NGRA framework protective for human health?

The primary objective of this evaluation was to understand if tier one of our DART NGRA framework provides sufficient protection for human health with respect to DART. To evaluate the protectiveness of the framework we compared the risk classifications assigned to each of the 49 chemical-exposure scenarios using traditional risk assessment methods, with the BER calculated by dividing the estimated internal exposure of the chemicals at the given external exposure scenario by the lowest PoD obtained from all NAMs (PoD from either HTTr, IPP, CSP, ReproTracker, devTOX quickPredict, H295R, or screening CALUX assay). The purpose of this comparison is to determine whether similar conclusions can be made on the risk of DART at a given chemical exposure in human, using the two different methods (i.e., traditional risk assessment methods using animal (and sometimes human) data, versus this novel NGRA approach). 17 of the 49 exposure scenarios are considered high risk for DART using traditional risk assessment methods. The optimal outcome of this evaluation would be for the NGRA framework to allow the identification of these same 17 exposure scenarios as high risk.

Conceptionally a BER greater than 1 indicates a low risk for the chemical at the given exposure, as bioactivity occurs at a higher concentration than the estimated internal Cmax value. Conversely, a BER of 1 or below suggests that bioactivity is expected at that exposure level. It should be stressed that a BER below 1 does not necessarily mean an adverse effect will occur, as the toolbox is measuring bioactivity which does not equate to adversity. Therefore, in practice, this NGRA approach uses the BER to identify chemical exposures with uncertain risk (BER <1), triggering additional evaluation before concluding on safety. This tiered approach ensures that potentially high-risk chemical exposures are captured and assessed in a protective manner. Ultimately the larger the BER calculated, the lower the risk to human health. Therefore, in our evaluation of the DART framework, we would expect all high-risk benchmark exposure scenarios to have BER values <1, and all low-risk benchmark exposure scenarios to obtain a BER >1.

**FIGURE 7:**
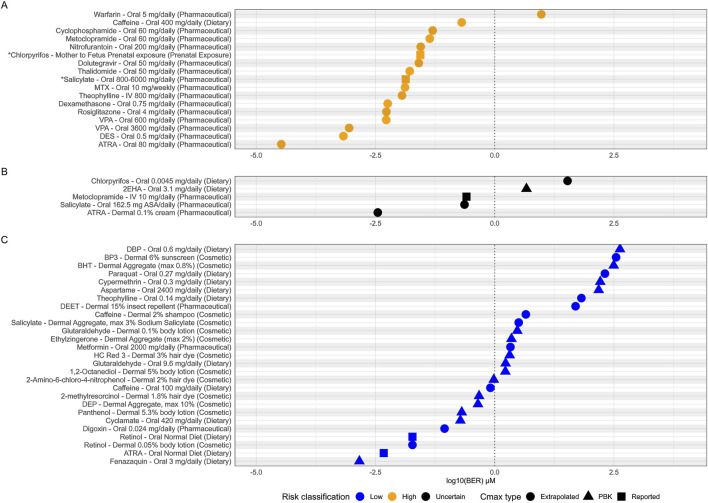
Estimated bioactivity exposure ratios (BERs) for each adult exposure scenario. **(A)** High risk scenarios (yellow), **(B)** uncertain risk scenarios (black) and **(C)** low risk scenarios (blue). BERs are plotted on a log10 scale and a conceptual BER threshold is shown by vertical dotted line at BER = 1. Points are shaped by the source of the Cmax used to calculate each BER. *Where no adult Cmax was available for ‘Salicylate–Oral 800-600 mg/daily’ and ‘Chloropyrifos- Mother to Fetus Prenatal’ exposures, BERs plotted are from the fetal exposure instead.

16 of the 17 (94%) high risk exposure scenarios, as determined by traditional risk assessment methods, had a BER of 1 or below (see Figure 7A). BERs do not materially change if non-pregnant, pregnant and fetal exposure is considered (see Supplementary Image 1). The one high risk exposure scenario which could not be identified as uncertain risk was the pharmaceutical use of warfarin (5 mg/daily/oral). The BER calculated for Warfarin was 9.5 indicating that this particular use would not result in any bioactivity and therefore, would be considered a low-risk exposure. This is a misclassification by the framework, as warfarin is a known developmental toxicant and is contraindicated in pregnancy at any exposure due to recognized patterns of major malformations (warfarin embryopathy), hemorrhage, an increased risk of spontaneous abortion and mortality (Hall et al., 1980; Stevenson et al., 1980).

Of the 27 low risk exposure scenarios, 16 (59%) were identified having a BER of above 1 and were therefore classified as low risk (Figure 7C) defining the utility of the framework. While tier one of the framework is designed to be protective to prevent harm, it must also be practical and distinguish true low-risk exposures if possible. However, some safe chemicals can show biological activity at exposure scenarios which are classified as safe to use. One of these exposure scenarios is low dietary caffeine intake (100 mg/daily, BER 0.8). This is considered a low-risk exposure for DART defined by the threshold established by EFSA for risks associated with both fetal growth restriction and late miscarriage and stillbirths (>200–300 mg/day) and corresponds to approximately one cup of coffee in a day (see Table 1 and Supplementary File S1). The lowest PoD used to calculate the BER of 0.8 comes from the Adenosine A2A receptor (5.2 μM), which is in the subfamily of receptors which promote caffeine ‘wakefulness’ effect. This example reflects the fact that bioactivity can drive pharmacological effects desired by the consumer (e.g., mental alertness following consumption of one caffeinated beverage) but that the degree of desired bioactivity seen at lower exposures does not necessarily lead to adversity. Further evaluation in subsequent tiers would aim to differentiate between the bioactivity seen at this low exposure which would be considered low risk and possible adversity seen for much higher exposures of caffeine which would be considered high risk.

For 5 of our 49 benchmark chemical exposure scenarios various regulatory authorities were unable to conclude on safety due to various degrees and sources of uncertainty in the traditional risk assessments, or differences in opinion between regulatory authorities (see Table 1 and Supplementary Data Sheet 1). Although not ‘true’ benchmarks for our evaluation (as no conclusion on high or low risk can be made) we were interested in comparing the outcome of our NGRA framework to these examples. Three of the five examples have BERs less than 1, which is expected for a true high-risk exposure (see Figure 7B). These are pharmaceutical use of metoclopramide (10 mg/daily/oral), pharmaceutical exposure to salicylate (via aspirin, 162.5 mg/daily/oral) and pharmaceutical use of ATRA (0.1% dermal). 2 of the 5 have BERs >1 which would be expected for any true low risk exposure, these included dietary intake of chlorpyrifos via pesticide residues (0.0045 mg/daily) and dietary intake of 2-ethylhexanoic acid as a flavoring (3.1 mg/daily).

### 3.6 How is protectiveness achieved?

**FIGURE 8:**
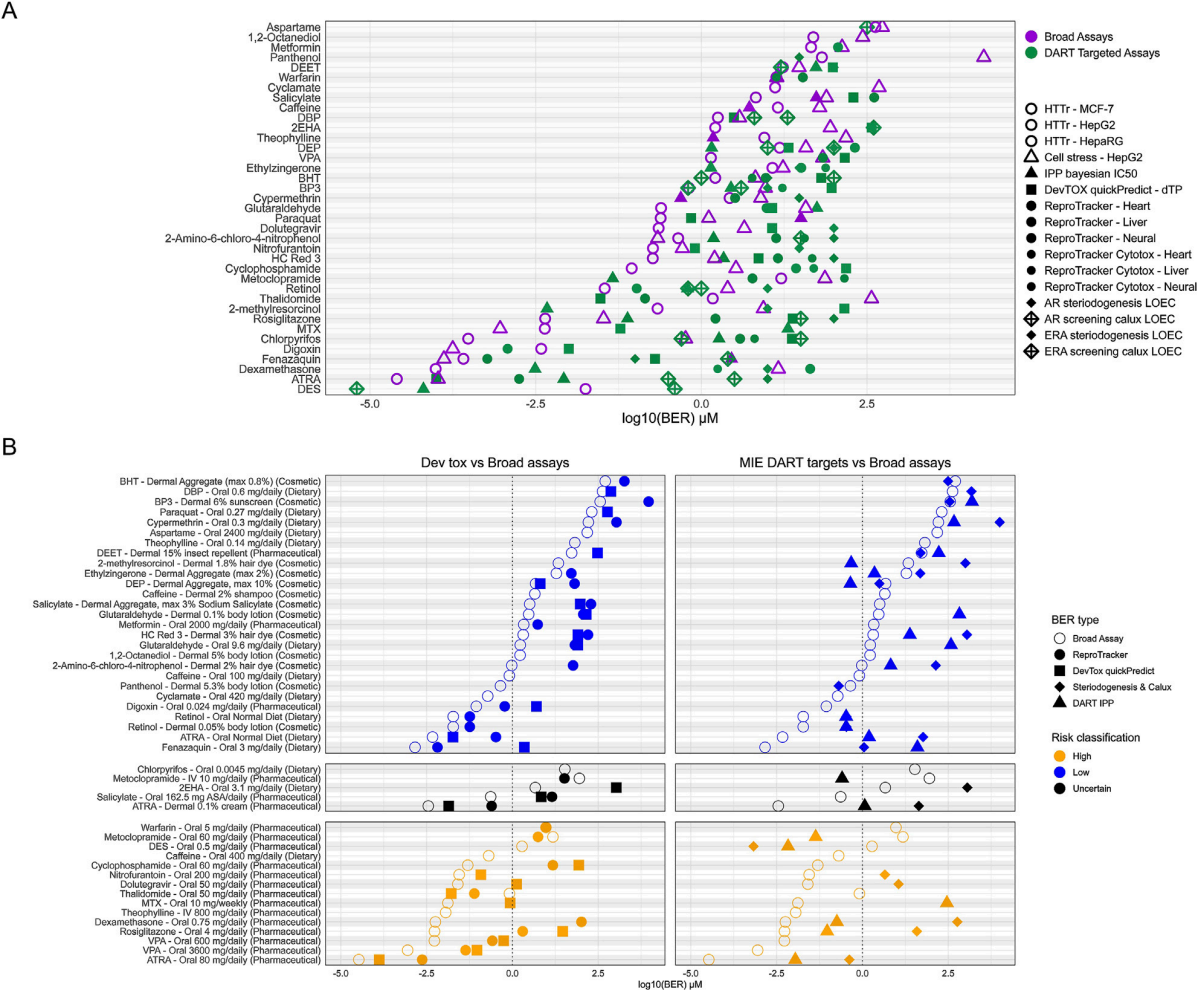
Distribution of PoDs and BERs from DART targeted NAMs versus broad screening tools. **(A)** Overview of lowest PoD from each NAM corresponding to dose -concentration calculations. NAMs are separated as DART targeted (green) versus broad screening (purple). IPP was split in two corresponding NAMs (see Supplementary File S5). **(B)** BERs for adult/non pregnant chemical exposure scenario for each compound as used before (see Figure 7). Shown are lowest BERs for each DART targeted NAM versus overall lowest BER from broad screening tools. Left panel shows DART targeted NAMs for developmental toxicity comprising of the ReproTracker and DevTox quickPredict assay and the right shows BERs of NAMs targeting DART MIEs including DART targeted IPP assays and both the steroidogenesis and Calux screening assays. Broad assay BER point are the BER derived from the lowest PoD from HTTr, cell stress panel (CSP) and broad IPP assays.

In addition to assessing overall protectiveness, we were further interested in determining whether both untargeted broad screening tools and targeted NAMs are necessary to achieve a protective approach for DART. We therefore separated tools into NAMs which are intended to detect DART- specific activity (from here on called DART-targeted NAMs) and broad screening tools. DART-targeted NAMs are DevTox quickPredict, ReproTracker, H295R, CALUX, and the IPP targets which could lead to DART specific effects (see Supplementary Data Sheet 5). The broad screening NAMs are the remaining IPP targets, the CSP and HTTr. The distribution of PoDs and BERs across the different NAMs was evaluated (see Figures 8A,B). For most compounds, the lowest PoD is achieved from broad screening tools (27/37), with HTTr most often generating the lowest PoD (20 compounds). For only 10 compounds a lowest PoD were derived from DART-targeted NAMs (see Figure 8A). Warfarin received equivalent lowest PoD concentrations from HTTr and ReproTracker with only 0.015 μM differences. Considering exposure and risk classification of the given exposure scenarios of the compounds, Metoclopramide, and DES are the only two compounds where protectiveness is achieved from DART-targeted NAMs alone (see Figure 8B). For both lowest PoD is achieved from receptor specific activity reflecting their specific mode of action (MoA), namely, estrogen activation and dopamine-2 receptor antagonism. For both compounds this receptor specific activity is thought to cause *in vivo* DART-related adverse events, namely, erectile disfunction for metoclopramide (Melis et al., 2022) and estrogen related reproductive effects for DES (Zilliacus et al., 2024). The only case where assays for screening for developmental toxicity show the lowest PoD is for Thalidomide (see Figure 8B). This finding is in line with the effects of thalidomide corresponding to perturbations in early embryonic development (Vargesson, 2022). For all other compounds with a high-risk exposure scenario a DART-targeted NAM can be found with a BER ≤ 1, indicating that at the given exposure, some form of DART-relevant bioactivity could occur. Altogether this data shows that a combination of DART-targeted and broad screening tools is needed to provide protectiveness for DART.

For most low-risk scenarios, BERs >1 can be found from DART-targeted NAMs, indicating low risk at the given exposure for DART. This is not unexpected, as many of the selected benchmark compounds show indications for DART at higher concentration *in vivo* as well (see Supplementary Data Sheet 1). For example, the tolerable daily intake (TDI) for dibutyl phthalate (DBP) is based on a NOAEL from developmental toxicity studies in rats; by using a safety factor for inter species differences of 200, concentrations below the TDI are judged to be safe for humans (see Table 1 and Supplementary Data Sheet 1). This is well reflected in the *in vitro* data where a positive response from devTox quickPredict and other *in vitro* data at concentrations above the TDI can be observed. For Retinol and ATRA bioactivity was detected within almost all NAMs at low concentrations leading to BERs <1 also for the low-risk scenario of normal dietary exposure to retinol and its metabolite. Vitamin A (retinol) and its metabolite is a crucial micronutrient especially needed in pregnancy. Deficiency during pregnancy is known to cause similar DART effects as seen for increased exposure above 3,000 μg RE/day *in vivo* (see Table 1 and Supplementary Data Sheet 1). Bioactivity of both substances are found at low concentrations which would indicate uncertain risk triggering further evaluation at higher tiers. BER <1 was also found for 2-methylresorcinol thyroid peroxidase (TPO) binding. TPO activity is known for resorcinol, a compound with a similar structure (Motonaga et al., 2016). For the low-risk exposure scenario for DEP, binding to the serotonin receptor HTR2B was seen with BER < 1. While no connection between the compound and the serotonin receptor could be established from literature, these findings would trigger higher tier testing in an NGRA approach. BERs below 1 for low-risk exposure scenarios for Fenazaquin, Digoxin and Panthenol are also unexpected and would also trigger higher tier testing.

## 4 Discussion

In recent years, substantial progress has been made in evaluating and adopting NGRA for human-relevant safety decision-making in systemic toxicity. This proof-of-concept study demonstrates that NGRA can be applied to DART, allowing for protective safety decisions concerning adults, pregnant women, and embryos to be made in a concentration-dependent manner in a first-tier assessment (see Figure 7; Supplementary Image 1). Using 37 benchmark compounds across 49 exposure scenarios, we achieved protectiveness for 17 out of 18 high-risk scenarios, with an overall framework utility of approximately 59%. Protectiveness was ensured through a combination of broad screening tools and targeted NAMs (see Figure 8).

Traditional risk assessment methods use data from several guideline studies to assess for systemic and DART effects throughout the entire reproductive cycle, using the lowest LOAEL/NOAEL to ensure overall human protection (Browne et al., 2024). As shown here, similar protection of human health can be achieved in a first-tier NGRA, providing that the selected NAMs offer the necessary biological coverage. The main difference is that while traditional risk assessments evaluate adverse effects such as malformations in pups, sperm motility, or maternal toxicity, NAMs used in NGRA focus on molecular initiating events (MIEs), early key events, or on general perturbations like transcriptional changes or stress responses. This means that while a first tier might not be predictive of DART effects in humans it can be protective, which is similar to the reality of the animal models we have previously relied on Browne et al. (2024). We are increasingly understanding from the high drug attrition rates in the pharmaceutical industry that effects in animal pre-clinical studies are not particularly predictive of clinical effects in humans (Weaver and Valentin, 2019; Pognan et al., 2023). An important point of consideration is that the same MIE’s or key events can lead to toxic effects in different tissues or in different life stages. For instance, many original IPP targets (Bowes et al., 2012) were selected to be protective for systemic toxicity in adults but are also crucial targets for reproduction and embryo development (see Supplementary Data Sheet 5). For example, targets relevant to the central nervous system, like the NMDA receptor or monoamine oxidase A, are also identified from the Integrated Chemical Environment (ICE) database as targets for developmental neurotoxicity. Therefore, using this example, if specific activity is detected, it could lead to adverse reactions in adults and/or the developing embryo, which might be dependent on exposure specifics (e.g., window of exposure or tissue specific accumulation/excretion) as well as tissue sensitivity. This means that while the MoA of a compound might lead to adaptive/reversible effects in an adult the same MoA could lead to an adverse reaction in the developing embryo or *vice versa*. General systemic effects in adults and DART specific effects can also occur at similar concentration which is often seen *in vivo* making separation of general systemic and DART specific effects difficult (Browne et al., 2024) and can lead to overlapping classification of substances (Worth and Berggren, 2024).

The finding that HTTr is most often the lowest PoD is not surprising, as perturbation of expression of DART-relevant genes and pathways, which could manifest as DART-related toxicity in a whole organism, can be identified in simple cell systems (Rajagopal et al., 2022; Janowska-Sejda et al., 2021). Transcriptomic analysis is able to identify known MoA for certain compounds associated, for example, with estrogen, glucocorticoid or retinoic acid activity in a dose dependent manner, resulting in protective PoDs (Harrill et al., 2024; Harrill et al., 2021; Basili et al., 2022). It is also known that maternal transcriptomic PODs are similar to fetal apical endpoint PODs *in vivo* (Johnson et al., 2022) showing similar protectiveness as compared with traditional risk assessments (Paul Friedman et al., 2020). While pathway or signature related analysis was out of scope for this work, one obvious example in our evaluation where data from transcriptomics analysis provides a protective PoD is VPA. VPA’s MoA for *in vivo* DART-related adverse events is at least partially thought to be caused by its histone deacetylase activity (LLoyd, 2013). Effects on local chromatin regions can result in improper expression of specific genes in different cell types (Park and Kim, 2020). As orchestrated transcriptional changes are a major part of iPSC differentiation and therefore successful embryo development, failure can cause dramatic effects which in the case of VPA can lead to craniofacial abnormalities and neural tube defects. The downstream consequences of transcriptional perturbation are detectable within the results of our framework evaluation with BERs <1 also coming from the ReproTracker and devTOX quickPredict assays (see Figure 8B). This demonstrates how using PoDs derived from transcriptomic data might reflect a more conservative approach addressing the same MoA.

Similarly, broad screening tools can conservatively detect the MoA for Methotrexate (MTX), a compound used for chemotherapeutic treatment, and a known human teratogen (Verberne et al., 2019), which can also lead to spontaneous abortion if taken within the first 8 weeks of pregnancy. MTX is a folic acid antagonist which can bind to dihydrofolate reductase, which converts dihydrofolate to tetrahydrofolate (van Gelder et al., 2010). Folate metabolism plays a pivotal role in various physiological processes like DNA biosynthesis, epigenetic maintenance, and redox defense (Ducker and Rabinowitz, 2017). By inhibiting DNA biosynthesis, MTX is expected to impact all proliferating cells. In line with this the lowest PoD for MTX derives from cell health measurements within the CSP, and the compound leads to cytotoxicity in proliferating iPSC cells in the devTOX quickPredict assay as well as within dose range finding experiments in ReproTracker at or below Cmax values. Folate metabolism also serves at the methyl donor for DNA methylation, an important factor for cell fate decision during early embryonic development (Breton-Larrivee et al., 2019). Inhibition thereby might be directly corelated to the teratogenic effects of MTX. While no effects on differentiation can be observed in ReproTracker this might be explained by the fact that cytotoxicity measurements are performed for 7 days in dose range finding experiments limiting the highest dose tested for the tri-lineage differentiation to 0.0085 μM. Additional testing in ReproTracker at concentration above 0.0085 μM also lead to effects on iPSC differentiation indicating potential embryotoxicity of the compound (data not shown). In comparison disruption to iPSC metabolism was detected in the devTOX quickPredict assay at 0.06 μM, resulting in a BER ∼1 (see Figure 8B).

The complex and numerous mechanisms leading to DART are still not well understood and are only known for a few well studied substances like VPA and MTX. Establishment and confidence in this mechanistic knowledge can take years or even decades for a single compound, as seen for Thalidomide, for which the teratogenic MoA was only determined in recent years despite decades of research. This might be even more challenging for compounds where adverse effects, such as reduced fetal or tissue-specific weight, might be caused as secondary effects. For example, changes in the morphology of the placenta could lead to malnutrition in the fetus, which, as a consequence, would result in reduced fetal weight. Therefore, using broad screening tools together with targeted assays (comprised of either a limited number of established targets for DART (IPP) and models of early embryonic development (ReproTracker and devTOX qP)), is one way of providing the broad biological coverage needed and presents an elegant solution to facilitate the replacement of animal testing for DART safety assessment now. We previously demonstrated 80% biological coverage of our DART tier 1 approach by comparing a marker list of genes involved in human reproduction and embryo-fetal development to the read-outs from our NAM toolbox (Rajagopal et al., 2022). This reduces slightly to ∼70% if the marker list is combined with the gene list identified in the DARTable genome (Janowska-Sejda et al., 2021) (data not shown). This is likely to increase after expanding to the ReProTracker gene base line expression over time which is not available. Identification of existing essential gaps are needed to achieve comprehensive protectiveness for DART. This could be achieved iteratively by further testing of additional benchmarks compounds. Identifying the MoA for benchmark chemicals exposures that the approach did not protect against will aid in discovering additional NAMs to be incorporated in future into tier 1. As an example, the current tier 1 framework did not identify pharmaceutical use of Warfarin as a high-risk scenario for DART. However, clinical data indicate that even the lowest recommended dose of Warfarin (5 mg daily), which we selected for our high-risk exposure scenario, can cause Warfarin embryopathy in humans (Sousa et al., 2018). Warfarin’s pharmacological mechanism of action involves inhibiting the vitamin K epoxide reductase complex, potentially leading to hemorrhage within the embryo and skeletal malformations which might be caused by defects in osteoblast differentiation (Jeong et al., 2011). While the ReproTracker assay flags potential embryotoxicity at higher concentrations, it is in the current state not sensitive enough to provide a protective PoD. Therefore, we initiated an additional osteoblast differentiation protocol. Preliminary data from this ongoing work shows lower PoD compared to the current ReproTracker analysis (Horcas Nieto et al., 2024). These preliminary findings are encouraging and suggest that including osteoblast lineage differentiation could enhance the protectiveness of the framework.

Safety frameworks must evolve over time, remaining flexible to incorporate new tests or NAMs or remove existing ones if they can be replaced by simpler, more cost-efficient, or more relevant systems. These systems should be adapted to meet upcoming needs and facilitate early decision-making for other regulatory or pre-regulatory uses, such as hazard labeling and classification, or within the context of safe and sustainable by design principles. While in this evaluation the DART targeted assays provide the most conservative PoDs for only a small number of substances, including them in the first tier may provide additional information for designing and refining subsequent tiers of the risk assessment in a hypothesis driven manner (see also (Paul Friedman et al., 2025)). This information can be obtained not only through *in vitro* results but also through *in silico* tools. Predictive *in silico* methods are increasingly recognized as alternatives to bridge the lack of knowledge about chemical properties and their biological activities (Mansouri et al., 2021). From our evaluation within this study the *in silico* models utilised show potential for providing useful information to incorporate into an NGRA for DART, what remains to be determined is a strategy in which *in silico* predictions can be generated and interpreted in tier 0 to better inform subsequent tiers of the framework and safety decision making process. Given the complexity of DART, the current battery of *in silico* models needs to be expanded in the near future covering other MIE’s, pathways and mechanisms important for DART. However, more well curated, high-quality data especially in the area of DART toxicity (*in vivo* and *in vitro*) is needed for further development of MoA specific models.

Furthermore, the evolution of these safety framework needs to go hand in hand with an evolving tiered approach to integrate population differences as well as DART subpopulation specific changes into exposure estimates. However, the available data on systemic exposure in those population groups is limited. For our benchmark chemicals, the quality and quantity of data varied considerably between chemicals and between different populations (see Figure 4) Whilst clinical PK data characterized by a clearly defined external dose and dosing schedule, and full time-course plasma concentration or as a minimum Cmax data was often available for exposures of non-pregnant study populations for pharmaceuticals, such data in general is not available for pregnant individuals and absent with regards to fetal exposures. There is a general lack of *in vivo* data for non-pharmaceuticals e.g., cosmetics or industrial chemicals. Data describing maternal and fetal exposures are in general less well-defined and less detailed and therefore carry greater uncertainties, e.g., Therapeutic Drug Monitoring (TDM) or Biomonitoring (BM) data can provide information regarding (assumed) steady state concentrations. However, it is in general not known in how far reported plasma concentrations are close to Cmax. For biomonitoring data, the external exposures resulting in the reported plasma concentration are usually not known and varied, hence this data can often only provide information on “typical” systemic exposures in selected populations. On some occasion, e.g., for nutrients or contaminants external exposures can be estimated from, e.g., dietary intake or product use information (e.g., for consumer products), or exposome studies (Cui et al., 2016). E.g., in the case of retinoic acid and retinol we used dietary intake data for retinol to relate to the reported biomonitoring data for retinol and ATRA. “Snapshots” of systemic exposure are available from poisoning cases or other case studies. However, this data as well is highly uncertain as dose and time from administration to hospitalization are often not exactly known, and high doses might be well outside the linear range of pharmacokinetics as enzyme and other physiological processes might get saturated.

The analysis of benchmark compounds with human-relevant clinical data (see Figure 6) indicates that exposure levels in non-pregnant populations are often similar to or higher than those in pregnant populations or fetuses. This suggests that general population exposure estimates could serve in most cases as conservative surrogate metrics for more specific sub-populations. However, instances such as Warfarin and Dexamethasone, where exposure levels in pregnant populations and fetuses seem to be higher than in non-pregnant populations, indicate that this may not always be the case. Therefore, it is important to understand when and to what extend physiological changes during pregnancy and specific transport mechanisms between the mother and the fetus might result in increased plasma concentrations to define criteria for when a more specific approach for the characterization of exposure is required.

In the absence of chemical-specific human *in vivo* data PBK modelling is often used to derive estimates of systemic concentrations based on parameterization of generic PBK models with chemical specific *in silico* and *in vitro* ADME parameters (Moxon et al., 2020; Cole et al., 2020). Often, in a first step, PBK simulations are performed for a “conservative” representative individual, e.g., in our case studies a 60 kg 30-year-old female, based on the assumption that a low body weight/size results in a comparatively higher Cmax for the same external dose. However, where we cannot be confident that exposure estimates for the general population are protective for DART-specific sub-populations more complex PBK models are required. These need to adequately represent the physiological changes occurring during pregnancy as well the physiology of the developing fetus and the ADME process involved in the transfer of chemicals between the mother and the fetus. Despite generic pregnancy PBK models being available in various PBK platforms (GastroPlus, SimCyp, PKSim, Httk, etc.) (Abduljalil et al., 2012; Chaphekar et al., 2021), model structures reflecting the embryo and fetus as well as placental transfer of chemicals differ significantly in their complexity. Whilst the more general and well understood physiological changes occurring during pregnancy are usually built into these models, processes that might more specifically impact on the biokinetics of some chemicals but not others, e.g., expression levels and changes of enzymes and especially transporters are often not, in particular with regards to DART relevant barriers and organs such as the placenta or blood-brain barrier. Chemical-specific knowledge and quantitative characterization of these process is required to extend the usefulness of PBK modelling beyond the generic.

Deterministic predictions of exposure in one (representative or “worst case”) individual can only be considered the starting point in the risk assessment process. Several studies collectively emphasize the need to consider population variability in toxicokinetic assessments to better protect public health (Dorne and Renwick, 2005). Hence, to account for the biokinetic variability within populations probabilistic PBK simulations should be performed in future to make predictions about distributions of exposures in populations of interest. For those simulations it must be defined what kind of variations and magnitudes of variability in physiology, product use and product formulations occur in the population. This, however, is currently still rather challenging to do. Other than with regards to general physiology, e.g., organ sizes, we often do not know and therefore cannot accurately parameterize the variability in metabolism, protein binding, differences in absorption, product formulations and use habits, and the impact of those on external and internal exposures. Therefore, PBK population simulations currently are unlikely to reflect the complete variability in a population. More understanding of the true variability of biokinetics and exposure in the population are needed to allow adequate parameterization of PBK models and development of uncertainty factors or other approaches to account for this uncertainty in model predictions. We found that for the majority of chemicals in our case study the TKVF calculated from the collated pharmacokinetic data is in good agreement with the uncertainty factor of 3.16 which is applied in traditional risk assessments, e.g., to the reference dose (RfD) or concentration (RfC) to account for inter-individual toxicokinetic variability with the objective to ensure that the derived exposure limits are protective of the most sensitive individuals in the population (Committee on Toxicity of Chemicals in Food, 2007). However, for some chemicals variability might be greater than this, e.g., as in the case of Nitrofurantoin (see Figure 5). For chemicals with unique toxicokinetic properties that differ significantly from the average, a more chemical specific uncertainty factor or more flexible approach for accounting for uncertainty is required for a more accurate and tailored assessment of risk. The challenge is–in the absence of *in vivo* data, i.e., in a NGRA setting, how to identify when a general uncertainty factor is not sufficient, and how to derive a more chemical specific estimate of uncertainty.

While our initial results show how a tier 1 NGRA framework can be protective for DART safety assessment further testing of more chemical exposures and additional NAM development (*in silico*, *in vitro*, and exposure) is essential to build confidence in this approach. It is particularly important to expand into already identified areas of interest where tools are missing (Sachana et al., 2019; Crofton and Mundy, 2021; Noyes et al., 2019) or where it is known that processes are still not well understood in human biology and species-specific differences are expected to make the approach more human relevant but also to account for different outcomes between human relevant NAMs and animal tests in future (Niethammer et al., 2022). To keep lower-tier approaches simple and affordable, it may be sufficient to add assays or *in silico* tools to measure or predict MIEs or early key events for toxicity, or tissue-specific transporters and metabolism in the first tier, while using the physiologically relevant system in a higher tier. However, there might be a transition period where higher-tier cell systems need to be integrated as a first-tier approach to accurately define the targets of interest before they can be replaced. This transition period is essential to validate that the identified targets are relevant and accurately reflect the underlying biological processes. Or to ensures that no additional targets are necessary within the first tier, as the desired level of protectiveness is already achieved. This process will help build confidence in the approach, demonstrating its robustness and reliability in various regulatory contexts. With this, it is important to continuously update and refine the NGRA approach as new data and insights become available. This iterative process will help to improve the accuracy and reliability of the risk assessments, ultimately leading to better protection of public health.

Moreover, collaboration between different scientific disciplines and stakeholders is essential to address the challenges and uncertainties in NGRA. By working together, we can develop more robust and comprehensive approaches (Fritsche et al., 2024). This collaborative effort will also facilitate the development of new methodologies and technologies that can enhance the predictive power of NGRA and ensure its applicability to a wide range of chemicals, exposure scenarios and regulatory uses.

## Data Availability

Raw experimental data from all assays are provided through the Dryad repository, available at: https://doi.org/10.5061/dryad.qz612jmt7

## Supplementary material

SUPPLEMENTARY DATA SHEET 1 Chemical exposure scenarios.

SUPPLEMENTARY DATA SHEET 2 Evaluation of *in silico* models.

SUPPLEMENTARY DATA SHEET 3 Overview of *in vivo* internal exposure for different DART subpopulations.

SUPPLEMENTARY DATA SHEET 4 PBK parameter summary.

SUPPLEMENTARY DATA SHEET 5 IPP assays overview.

SUPPLEMENTARY DATA SHEET 6 Conc Dose Ratios.
