# Supplementary material for: An advancement in developmental and reproductive toxicity (DART) risk assessment: evaluation of a bioactivity and exposure-based NAM toolbox
Source: Front Toxicol. 2025 Jun 30;7:1602065. doi: 10.3389/ftox.2025.1602065 (PMC12256496; doi:10.3389/ftox.2025.1602065)
Supplement: Supplementary file 3 [file DataSheet3.docx]

**Supplementary File S3**

In vivo pharmacokinetic data were used to establish the relationship between external and internal dose. Scatterplots show reported external doses and resulting measured plasma, serum or cord blood concentrations. The underlying data can be found within the references given. Data is reported for non-pregnant (blue), pregnant (orange) and foetal (green) population subgroups. Marker shape symbolises the route of administration.

**Valproic Acid**


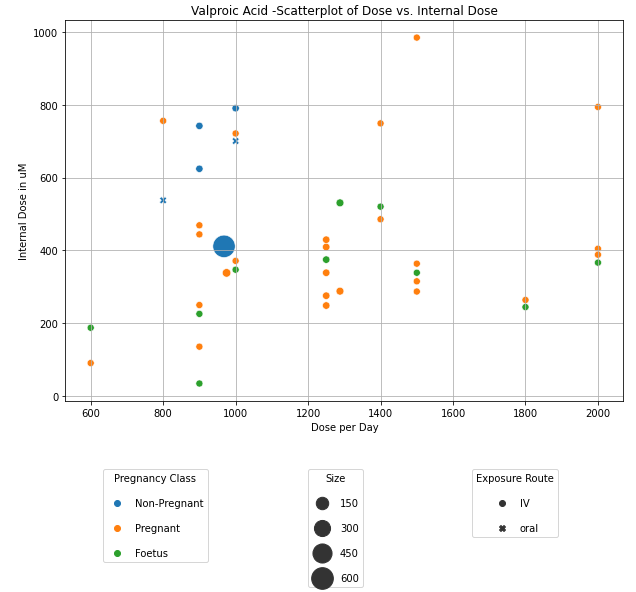


References

[1-8]

**Theophylline**


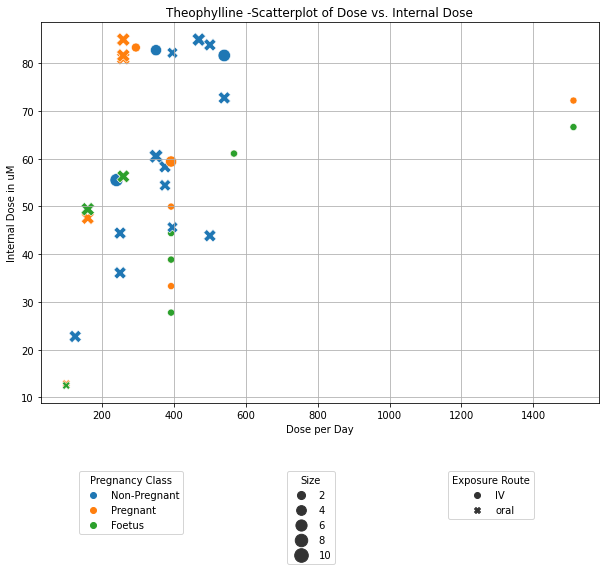


References

[9-20]

**Salicylate**


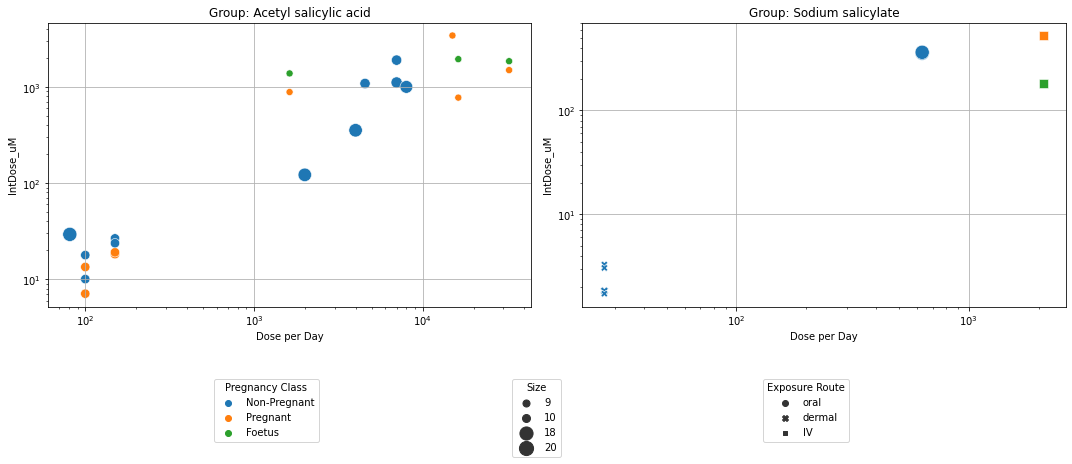


References

[21-31]

**All-trans-Retinoic Acid**


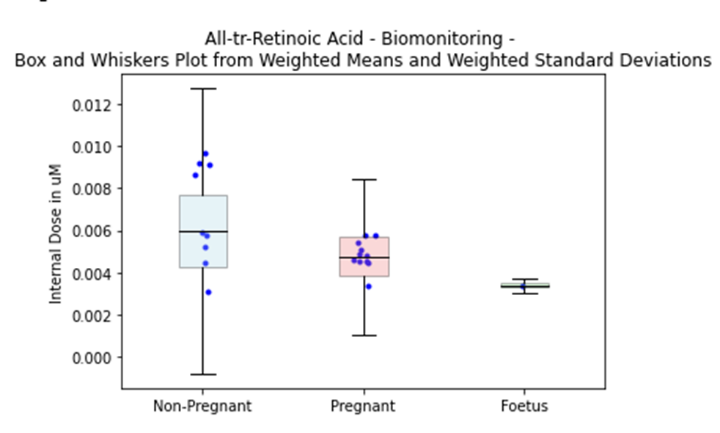

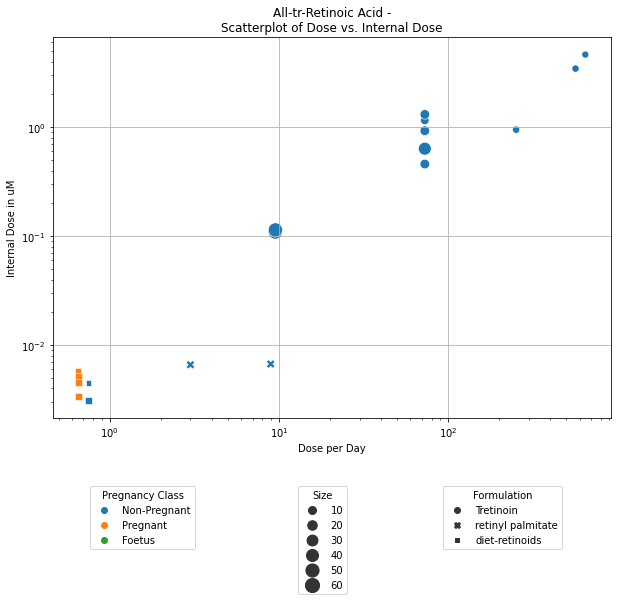


References

[32-43]

**Retinol**


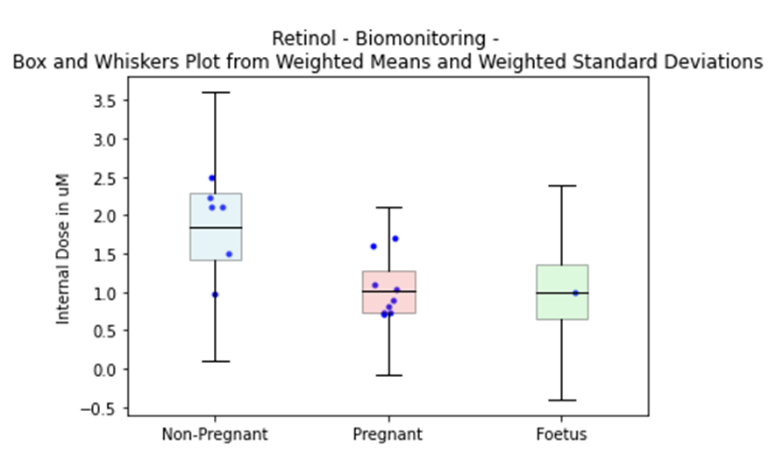

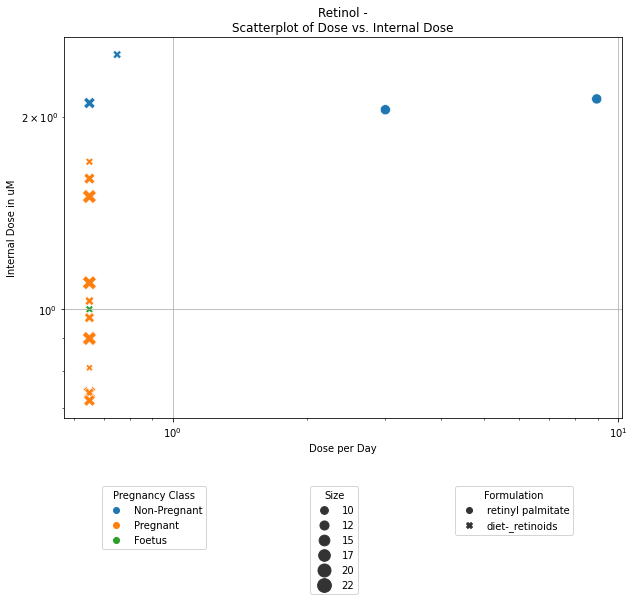


References

[33, 34, 36, 41, 42]

**Caffeine**


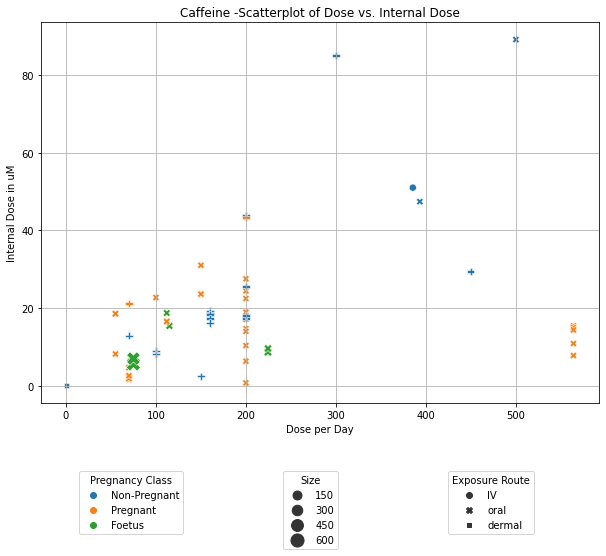


References

[44-60]

**Rosiglitazone**


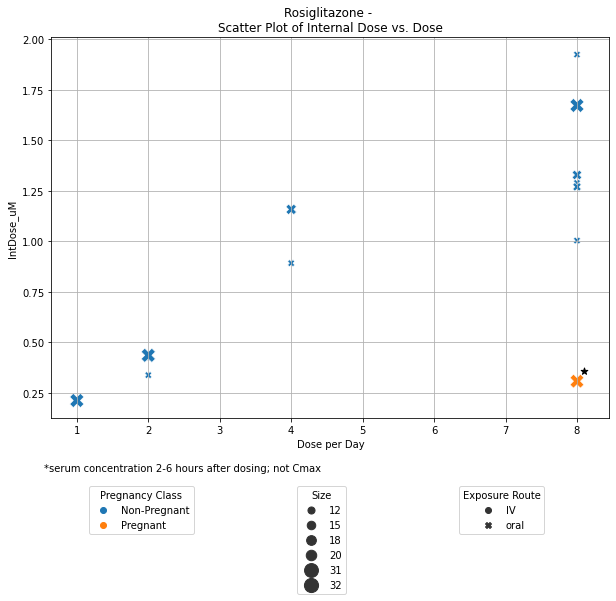


References

[61-66]

**Dolutegravir**


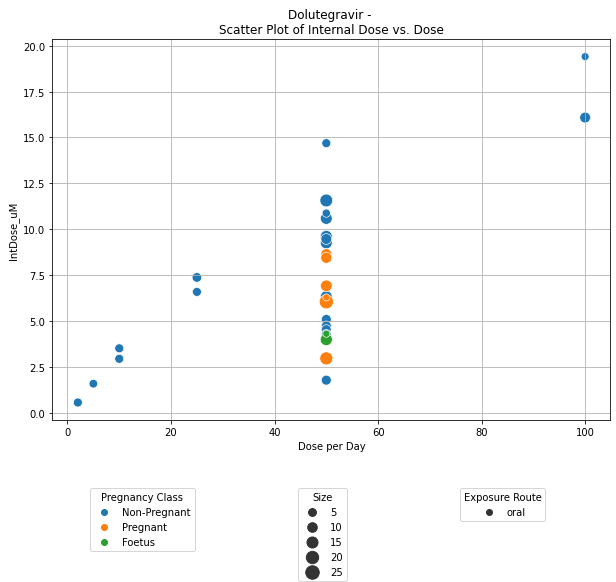


References

[67-73]

**Paraquat**


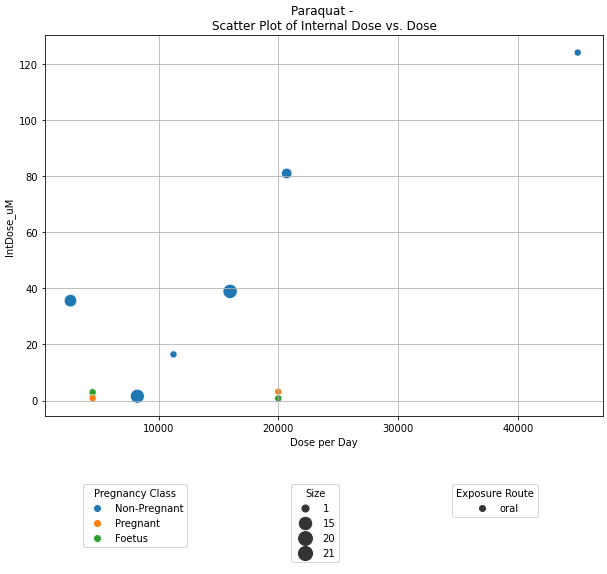


References

[74-79]

**Digoxin**


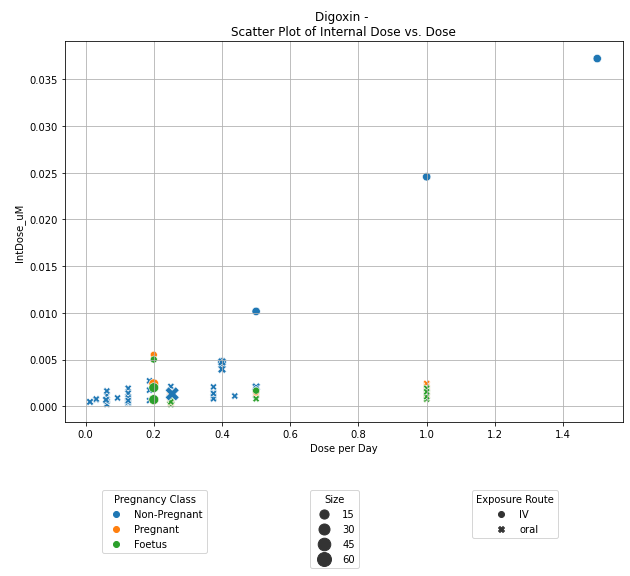


References

[80-84]

**Nitrofurantoin**


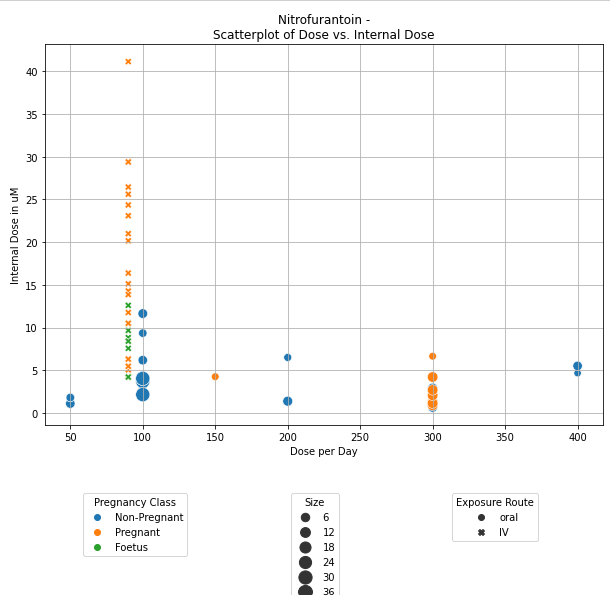


References

[85-90]

**Metoclopramide**


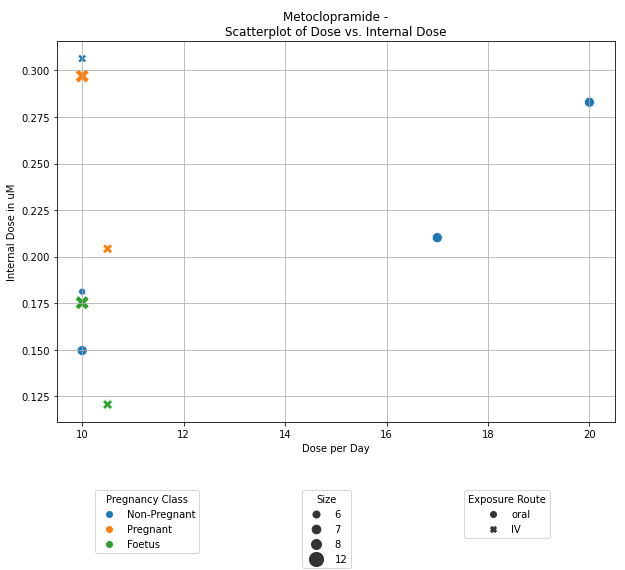


References

[91-95]

**Metformin**


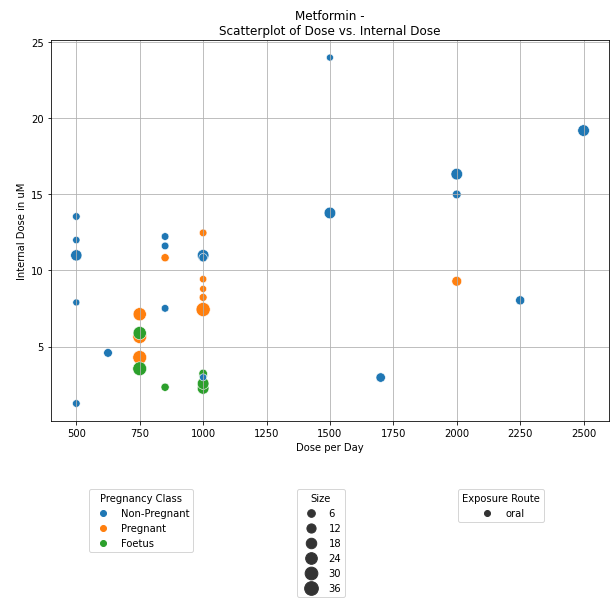


References

[96-105]

**Methotrexate**


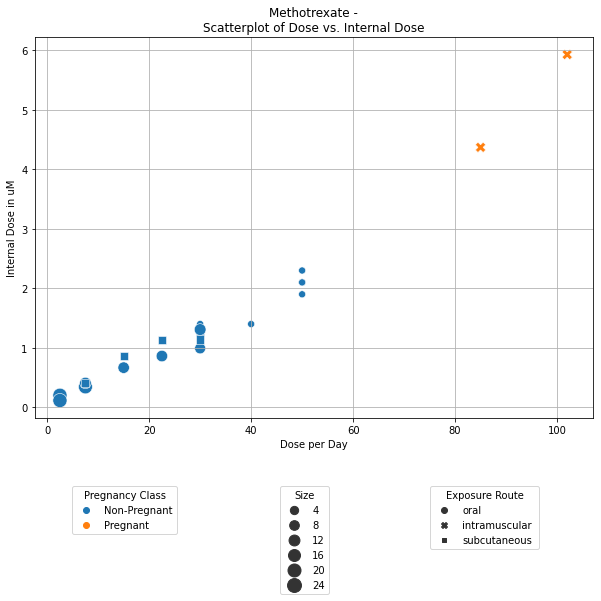


[106-112]

**Cyclophosphamide**


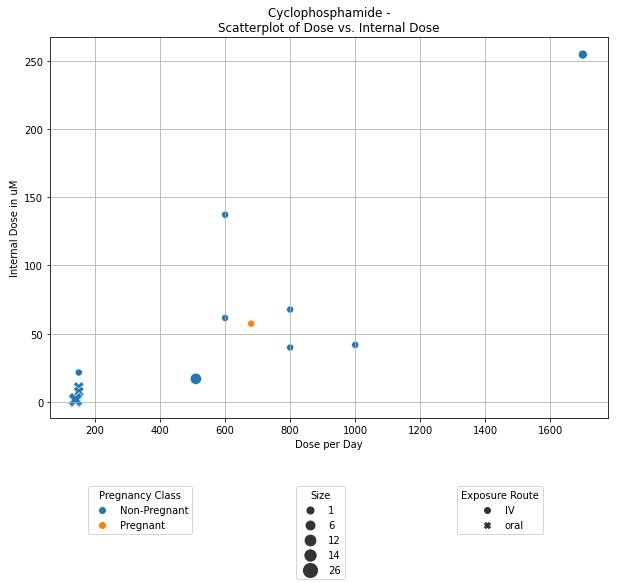


References

[113-118]

**Warfarin**


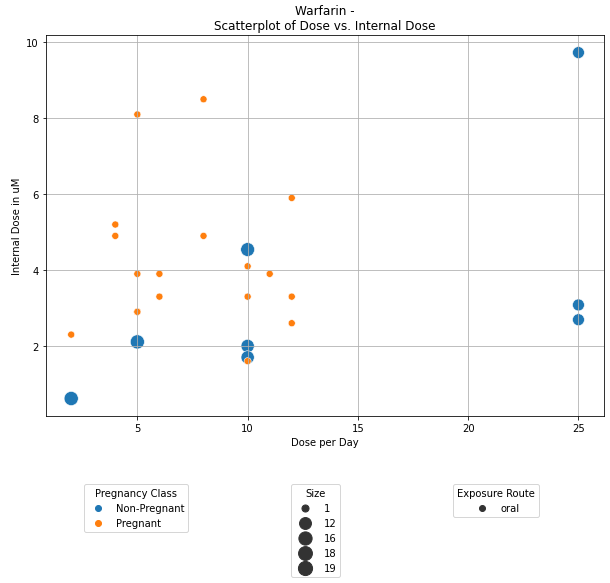


References

[119-123]

**Thalidomide**


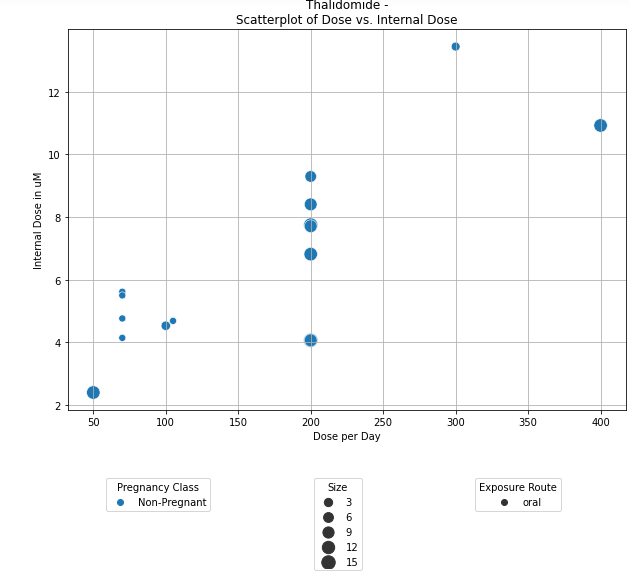


References

[124-129]

**Benzphenone-3**


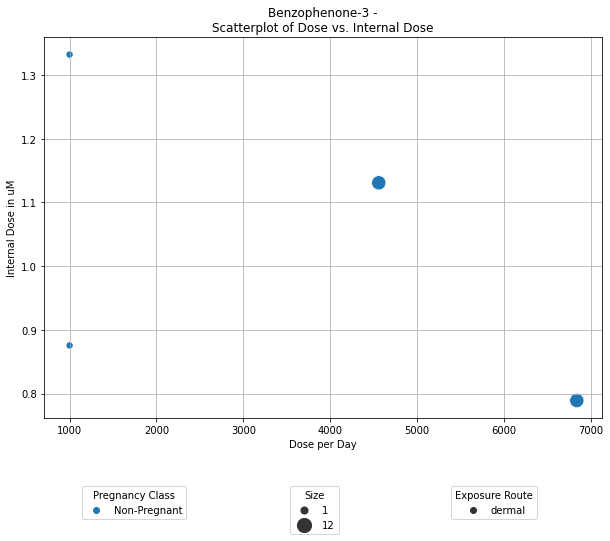


References

[130, 131]

**Diethyl stilbestrol**


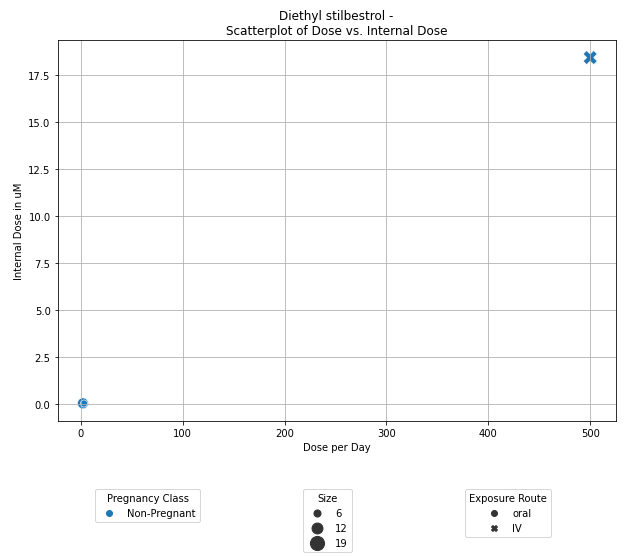


References

[132-134]

**Dexamethasone**


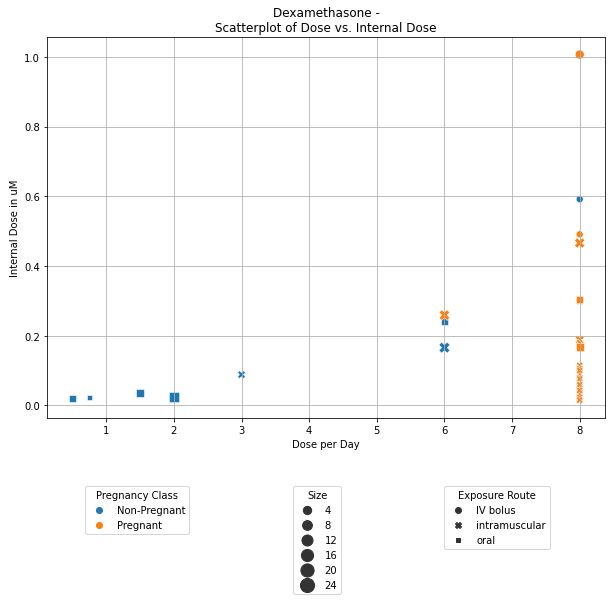


References

[135-140]

**BHT**


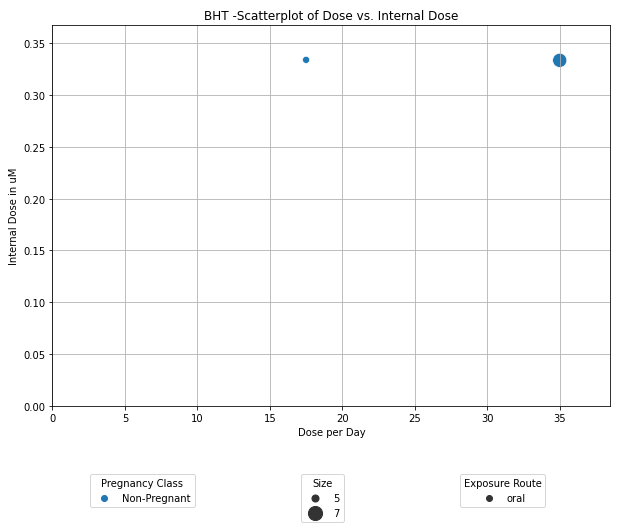


References

[141]

**DEET**


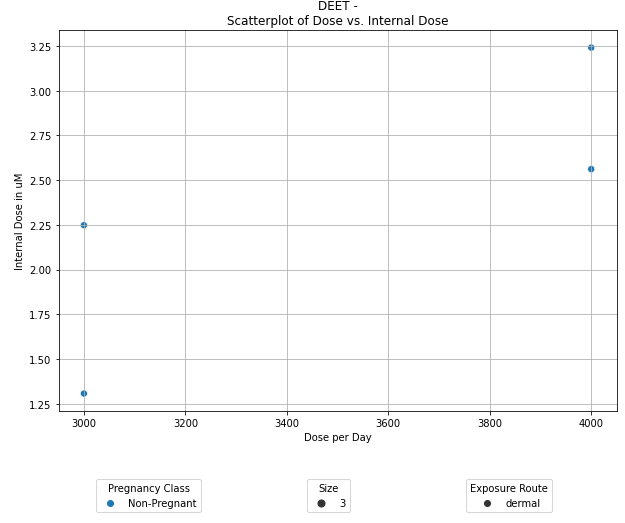


[142]

**Dibutyl Phthalate (DBP) and Diethyl Phthalate (DEP)** - Biomonitoring Data


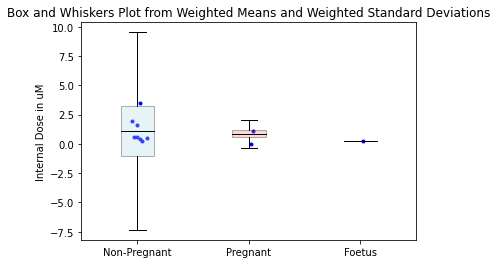

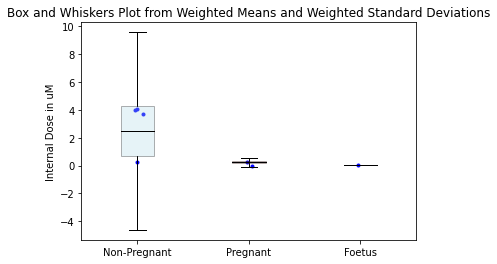


DBP

DEP

References

[143-148]

**Chlorpyrifos** - Biomonitoring Data


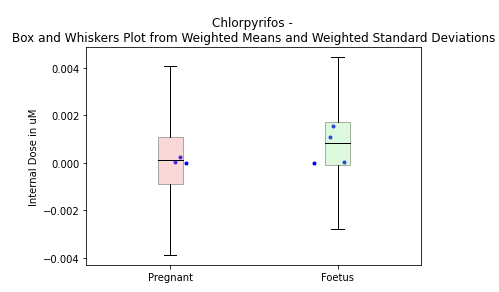


References

[149-152]

References

1. Jäger-Roman, E., et al., *Fetal growth, major malformations, and minor anomalies in infants born to women receiving valproic acid.* The Journal of Pediatrics, 1986. **108**(6): p. 997-1004.

2. Johannessen Landmark, C., et al., *Pharmacokinetic variability of valproate in women of childbearing age.* Epilepsia, 2017. **58**(10): p. e142-e146.

3. Johannessen Landmark, C., et al., *Pharmacokinetic variability of valproate during pregnancy – Implications for the use of therapeutic drug monitoring.* Epilepsy Research, 2018. **141**: p. 31-37.

4. Koerner, M., et al., *Valproic Acid Disposition and Protein Binding in Pregnancy.* Therapeutic Drug Monitoring, 1989. **11**(3): p. 228-230.

5. Nau, H., H. Helge, and W. Luck, *Valproic acid in the perinatal period: Decreased maternal serum protein binding results in fetal accumulation and neonatal displacement of the drug and some metabolites.* The Journal of Pediatrics, 1984. **104**(4): p. 627-634.

6. Nitsche, V. and H. Mascher, *The Pharmacokinetics of Valproic Acid After Oral and Parenteral Administration in Healthy Volunteers.* Epilepsia, 1982. **23**(2): p. 153-162.

7. Perucca, E., et al., *Disposition of sodium valproate in epileptic patients.* British Journal of Clinical Pharmacology, 1978. **5**(6): p. 495-499.

8. Tsuru, N., T. Maeda, and M. Tsuruoka, *Three Cases of Delivery under Sodium Valproate—Placental Transfer, Milk Transfer and Probable Teratogenicity of Sodium Valproate—.* Psychiatry and Clinical Neurosciences, 1988. **42**(1): p. 89-96.

9. Abduljalil, K., I. Gardner, and M. Jamei, *Application of a Physiologically Based Pharmacokinetic Approach to Predict Theophylline Pharmacokinetics Using Virtual Non-Pregnant, Pregnant, Fetal, Breast-Feeding, and Neonatal Populations.* Frontiers in Pediatrics, 2022. **10**.

10. Arwood, L.L., J.F. Dasta, and C. Friedman, *Placental transfer of theophylline: two case reports.* Pediatrics, 1979. **63**(6): p. 844-6.

11. Brazier, J.L. and B. Salle, *Conversion of theophylline to caffeine by the human fetus.* Semin Perinatol, 1981. **5**(4): p. 315-20.

12. Jonkman, J.H., et al., *Chronopharmacokinetics of theophylline after sustained release and intravenous administration to adults.* Eur J Clin Pharmacol, 1984. **26**(2): p. 215-22.

13. Jonkman, J.H.G., et al., *Disposition and clinical pharmacokinetics of theophylline after administration of a new sustained release tablet.* European Journal of Clinical Pharmacology, 1981. **21**(1): p. 39-44.

14. Jonkman, J.H.G., et al., *Disposition and clinical pharmacokinetics of microcrystalline theophylline.* European Journal of Clinical Pharmacology, 1980. **17**(5): p. 379-384.

15. Labovitz, E. and S. Spector, *Placental Theophylline Transfer in Pregnant Asthmatics.* JAMA, 1982. **247**(6): p. 786-788.

16. Romero, R., et al., *Pharmacokinetics of intravenous theophylline in pregnant patients at term.* Am J Perinatol, 1983. **1**(1): p. 31-5.

17. Ron, M., et al., *Maternal-Fetal Transfer of Aminophylline.* Acta Obstetricia et Gynecologica Scandinavica, 1984. **63**(3): p. 217-218.

18. Rovei, V., F. Chanoine, and S.M. Benedetti, *Pharmacokinetics of theophylline: a dose-range study.* British Journal of Clinical Pharmacology, 1982. **14**(6): p. 769-778.

19. Stec, G.P., et al., *Kinetics of theophylline transfer to breast milk.* Clinical Pharmacology & Therapeutics, 1980. **28**(3): p. 404-408.

20. Torrent, J., et al., *Theophylline pharmacokinetics following single and repeated administration of slow-release capsules.* European Journal of Drug Metabolism and Pharmacokinetics, 1988. **13**(4): p. 225-230.

21. *2001 North American Congress of Clinical Toxicology Annual Meeting.* Journal of Toxicology: Clinical Toxicology, 2001. **39**(5): p. 455-568.

22. Bochner, F., et al., *Salicyl phenolic glucuronide pharmacokinetics in patients with rheumatoid arthritis.* European Journal of Clinical Pharmacology, 1987. **32**(2): p. 153-158.

23. Davis, D.A.P., et al., *Percutaneous Absorption of Salicylic Acid after Repeated (14-day) In Vivo Administration to Normal, Acnegenic or Aged Human Skin.* Journal of Pharmaceutical Sciences, 1997. **86**(8): p. 896-899.

24. Elis, J., et al., *The distribution of sodium salicylate in the human fetus.* Int J Clin Pharmacol Biopharm, 1978. **16**(8): p. 365-7.

25. Farid, H., M.H. Wojcik, and K.B. Christopher, *A 19-year-old at 37 weeks gestation with an acute acetylsalicylic acid overdose.* NDT Plus, 2011. **4**(6): p. 394-396.

26. Garrettson, L.K., J.A. Procknal, and G. Levy, *Fetal acquisition and neonatal elimination of a large amount of salicylate.* Clinical Pharmacology & Therapeutics, 1975. **17**(1): p. 98-103.

27. Gibson, T., et al., *Kinetics of salicylate metabolism.* British Journal of Clinical Pharmacology, 1975. **2**(3): p. 233-238.

28. Miaskiewicz, S.L., C.A. Shively, and E.S. Vesell, *Sex differences in absorption kinetics of sodium salicylate.* Clinical Pharmacology & Therapeutics, 1982. **31**(1): p. 30-37.

29. Palatnick, W. and M. Tenenbein, *Aspirin Poisoning During Pregnancy: Increased Fetal Sensitivity.* Am J Perinatol, 1998. **15**(01): p. 39-41.

30. Rivera, W., et al., *Delayed Salicylate Toxicity at 35 Hours Without Early Manifestations Following a Single Salicylate Ingestion.* Annals of Pharmacotherapy, 2004. **38**(7-8): p. 1186-1188.

31. Shanmugalingam, R., et al., *A pharmacokinetic assessment of optimal dosing, preparation, and chronotherapy of aspirin in pregnancy.* American Journal of Obstetrics and Gynecology, 2019. **221**(3): p. 255.e1-255.e9.

32. Arnold, S.L.M., et al., *A sensitive and specific method for measurement of multiple retinoids in human serum with UHPLC-MS/MS.* Journal of Lipid Research, 2012. **53**(3): p. 587-598.

33. Berggren Söderlund, M., G.A. Fex, and P. Nilsson-Ehle, *Concentrations of retinoids in early pregnancy and in newborns and their mothers2.* The American Journal of Clinical Nutrition, 2005. **81**(3): p. 633-636.

34. Czuba, L.C., et al., *Plasma Retinoid Concentrations Are Altered in Pregnant Women.* Nutrients, 2022. **14**(7): p. 1365.

35. IV, H.C.P., et al., *all-trans Retinoic Acid: a Dose-Seeking Study in Solid Tumors.* Annals of the New York Academy of Sciences, 1993. **691**(1): p. 250-252.

36. Jeong, H., et al., *Temporal changes in the systemic concentrations of retinoids in pregnant and postpartum women.* PLOS ONE, 2023. **18**(2): p. e0280424.

37. Latriano, L., et al., *The percutaneous absorption of topically applied tretinoin and its effect on endogenous concentrations of tretinoin and its metabolites after single doses or long-term use.* Journal of the American Academy of Dermatology, 1997. **36**(3, Supplement): p. S37-S46.

38. Lee, J.S., et al., *Phase I evaluation of all-trans-retinoic acid in adults with solid tumors.* Journal of Clinical Oncology, 1993. **11**(5): p. 959-966.

39. Muindi, J.R., et al., *Clinical pharmacology of oral all-trans retinoic acid in patients with acute promyelocytic leukemia.* Cancer Res, 1992. **52**(8): p. 2138-42.

40. Muindi, J.R., C.W. Young, and R.P. Warrell, Jr., *Clinical pharmacology of all-trans retinoic acid.* Leukemia, 1994. **8**(11): p. 1807-12.

41. Nohynek, G.J., et al., *Repeated topical treatment, in contrast to single oral doses, with Vitamin A-containing preparations does not affect plasma concentrations of retinol, retinyl esters or retinoic acids in female subjects of child-bearing age.* Toxicology Letters, 2006. **163**(1): p. 65-76.

42. Söderlund, M., et al., *Biological variation of retinoids in man.* Scandinavian Journal of Clinical and Laboratory Investigation, 2002. **62**(7): p. 511-519.

43. Thudi, N.R., et al., *Pharmacokinetic and bioequivalence study of endogenous compound tretinoin 10 mg capsules in healthy volunteers by base line correction approach.* Clinical Research and Regulatory Affairs, 2011. **28**(3): p. 68-73.

44. Birkett, D. and J. Miners, *Caffeine renal clearance and urine caffeine concentrations during steady state dosing. Implications for monitoring caffeine intake during sports events.* British Journal of Clinical Pharmacology, 1991. **31**(4): p. 405-408.

45. Blanchard, J. and S.J.A. Sawers, *The absolute bioavailability of caffeine in man.* European Journal of Clinical Pharmacology, 1983. **24**(1): p. 93-98.

46. Brazier, J.L., et al., *Pharmacokinetics of Caffeine during and after Pregnancy.* Developmental Pharmacology and Therapeutics, 2017. **6**(5): p. 315-322.

47. Chen, Y., et al., *Genistein alters caffeine exposure in healthy female volunteers.* European Journal of Clinical Pharmacology, 2011. **67**(4): p. 347-353.

48. Darakjian, L.I. and A. Kaddoumi, *Physiologically Based Pharmacokinetic/Pharmacodynamic Model for Caffeine Disposition in Pregnancy.* Molecular Pharmaceutics, 2019. **16**(3): p. 1340-1349.

49. Devoe, L.D., et al., *Maternal caffeine consumption and fetal behavior in normal third-trimester pregnancy.* American Journal of Obstetrics and Gynecology, 1993. **168**(4): p. 1105-1110.

50. Grosso, L.M., et al., *Prenatal Caffeine Assessment: Fetal and Maternal Biomarkers or Self-Reported Intake?* Annals of Epidemiology, 2008. **18**(3): p. 172-178.

51. Hammami, M.M. and S.N. Alvi, *Large Intra-subject Variability in Caffeine Pharmacokinetics: Randomized Cross-over Study of Single Caffeine Product.* Drug Res (Stuttg), 2017. **67**(09): p. 539-546.

52. Kaplan, G.B., et al., *Dose-Dependent Pharmacokinetics and Psychomotor Effects of Caffeine in Humans.* The Journal of Clinical Pharmacology, 1997. **37**(8): p. 693-703.

53. Morde, A., et al., *Pharmacokinetic profile of a novel sustained-release caffeine with extended benefits on alertness and mood: A randomized, double-blind, single-dose, active-controlled, crossover study.* Current Research in Behavioral Sciences, 2021. **2**: p. 100036.

54. Oo, C.Y., et al., *Pharmacokinetics of Caffeine and Its Demethylated Metabolites in Lactation: Predictions of Milk to Serum Concentration Ratios.* Pharmaceutical Research, 1995. **12**(2): p. 313-316.

55. Otberg, N., et al., *The role of hair follicles in the percutaneous absorption of caffeine.* British Journal of Clinical Pharmacology, 2008. **65**(4): p. 488-492.

56. Stavchansky, S., et al., *Pharmacokinetics of caffeine in breast milk and plasma after single oral administration of caffeine to lactating mothers.* Biopharmaceutics & Drug Disposition, 1988. **9**(3): p. 285-299.

57. Van't Hoff, W., *CAFFEINE IN PREGNANCY.* The Lancet, 1982. **319**(8279): p. 1020.

58. Wang, Y.-H., et al., *Development of a liquid chromatography-tandem mass spectrometry (LC–MS/MS) method for characterizing caffeine, methylliberine, and theacrine pharmacokinetics in humans.* Journal of Chromatography B, 2020. **1155**: p. 122278.

59. White Jr, J.R., et al., *Pharmacokinetic analysis and comparison of caffeine administered rapidly or slowly in coffee chilled or hot versus chilled energy drink in healthy young adults.* Clinical Toxicology, 2016. **54**(4): p. 308-312.

60. Yu, T., et al., *Pregnancy-induced changes in the pharmacokinetics of caffeine and its metabolites.* The Journal of Clinical Pharmacology, 2016. **56**(5): p. 590-596.

61. Chan, L.Y.-S., J.H.-k. Yeung, and T.K. Lau, *Placental transfer of rosiglitazone in the first trimester of human pregnancy.* Fertility and Sterility, 2005. **83**(4): p. 955-958.

62. Chapelsky, M.C., et al., *Pharmacokinetics of Rosiglitazone in Patients with Varying Degrees of Renal Insufficiency.* The Journal of Clinical Pharmacology, 2003. **43**(3): p. 252-259.

63. Freed, M.I., et al., *Systemic exposure to rosiglitazone is unaltered by food.* European Journal of Clinical Pharmacology, 1999. **55**(1): p. 53-56.

64. Miller, A.K., R.A. DiCicco, and M.I. Freed, *The effect of ranitidine on the pharmacokinetics of rosiglitazone in healthy adult male volunteers.* Clinical Therapeutics, 2002. **24**(7): p. 1062-1071.

65. Suresh Kumar, J.N., et al., *Effect of ciprofloxacin and ibuprofen on thein vitro Metabolism of rosiglitazone and oral pharmacokinetics of rosiglitazone in healthy human volunteers.* European Journal of Drug Metabolism and Pharmacokinetics, 2008. **33**(4): p. 237-242.

66. Yu, J., et al., *Bioequivalence and comparison of pharmacokinetic properties of 4-mg tablet formulations of rosiglitazone hydrochloride and rosiglitazone maleate: A single-dose, randomized, open-label, two-period crossover study in healthy adult male Chinese volunteers.* Clinical Therapeutics, 2008. **30**(12): p. 2272-2279.

67. Min, S., et al., *Pharmacokinetics and safety of S/GSK1349572, a next-generation HIV integrase inhibitor, in healthy volunteers.* Antimicrob Agents Chemother, 2010. **54**(1): p. 254-8.

68. Mulligan, N., et al., *Dolutegravir pharmacokinetics in pregnant and postpartum women living with HIV.* Aids, 2018. **32**(6): p. 729-737.

69. Rimawi, B.H., et al., *Pharmacokinetics and Placental Transfer of Elvitegravir, Dolutegravir, and Other Antiretrovirals during Pregnancy.* Antimicrobial Agents and Chemotherapy, 2017. **61**(6): p. 10.1128/aac.02213-16.

70. Song, I., et al., *Pharmacokinetics of dolutegravir when administered with mineral supplements in healthy adult subjects.* The Journal of Clinical Pharmacology, 2015. **55**(5): p. 490-496.

71. Song, I., et al., *Effect of food on the pharmacokinetics of the integrase inhibitor dolutegravir.* Antimicrob Agents Chemother, 2012. **56**(3): p. 1627-9.

72. Waitt, C., et al., *Safety and pharmacokinetics of dolutegravir in pregnant mothers with HIV infection and their neonates: A randomised trial (DolPHIN-1 study).* PLoS Med, 2019. **16**(9): p. e1002895.

73. Wang, X., et al., *Pharmacokinetics of dolutegravir 100 mg once daily with rifampicin.* International Journal of Antimicrobial Agents, 2019. **54**(2): p. 202-206.

74. Chen, J., et al., *Fetal outcomes after intentional ingestion of paraquat: A case report.* Medicine (Baltimore), 2020. **99**(1): p. e18136.

75. Houzé, P., et al., *Toxicokinetics of Paraquat in Humans.* Human & Experimental Toxicology, 1990. **9**(1): p. 5-12.

76. Kim, H.-J., et al., *Toxicokinetics of paraquat in Korean patients with acute poisoning.* kjpp, 2015. **20**(1): p. 35-39.

77. Raynal, P., A.E. Bossard, and G. Carles, *Intoxication au paraquat en début de grossesse.* Gynécologie Obstétrique & Fertilité, 2003. **31**(5): p. 449-451.

78. Seok, S., et al., *The Time between Paraquat Ingestion and a Negative Dithionite Urine Test in an Independent Risk Factor for Death and Organ Failure in Acute Paraquat Intoxication.* J Korean Med Sci, 2012. **27**(9): p. 993-998.

79. Shi, L., et al., *The toxicokinetics of acute paraquat poisoning in specific patients: a case series.* Journal of International Medical Research, 2022. **50**(9): p. 03000605221122745.

80. Azancot-Benisty, A., et al., *Clinical and pharmacologic study of fetal supraventricular tachyarrhythmias.* The Journal of Pediatrics, 1992. **121**(4): p. 608-613.

81. Lloyd, B.L., et al., *Pharmacokinetics and bioavailability of digoxin capsules, solution and tablets after single and multiple doses.* The American Journal of Cardiology, 1978. **42**(1): p. 129-136.

82. Nagashima, M., et al., *Intrauterine supraventricular tachyarrhythmias and transplacental digitalisation.* Archives of Disease in Childhood, 1986. **61**(10): p. 996-1000.

83. Ochs, H.R., et al., *Dose-independent pharmacokinetics of digoxin in humans.* Am Heart J, 1978. **96**(4): p. 507-11.

84. Okada, R.D., et al., *Relationship between plasma concentration and dose of digoxin in patients with and without renal impairment.* Circulation, 1978. **58**(6): p. 1196-1203.

85. Huttner, A., et al., *The pharmacokinetics of nitrofurantoin in healthy female volunteers: a randomized crossover study.* Journal of Antimicrobial Chemotherapy, 2019. **74**(6): p. 1656-1661.

86. Nöschel, H., et al., *[Studies on the kinetics of nitrofurantoin (Nifurantin) in normal pregnancy, gestoses, pyelonephritis and labor (author's transl)].* Pharmazie, 1982. **37**(3): p. 204-5.

87. Perry, J.E. and A.L. Leblanc, *Transfer of nitrofurantoin across the human placenta.* Tex Rep Biol Med, 1967. **25**(2): p. 265-9.

88. Pons, G., et al., *Nitrofurantoin excretion in human milk.* Dev Pharmacol Ther, 1990. **14**(3): p. 148-52.

89. Sharma, R.P., E.J. Burgers, and J.B. Beltman, *Development of a Physiologically Based Pharmacokinetic Model for Nitrofurantoin in Rabbits, Rats, and Humans.* Pharmaceutics, 2023. **15**(9): p. 2199.

90. Wijma, R.A., et al., *Review of the pharmacokinetic properties of nitrofurantoin and nitroxoline.* Journal of Antimicrobial Chemotherapy, 2018. **73**(11): p. 2916-2926.

91. Arvela, P., et al., *Placental transfer and hormonal effects of metoclopramide.* European Journal of Clinical Pharmacology, 1983. **24**(3): p. 345-348.

92. Bateman, D., C. Kahn, and D. Davies, *The pharmacokinetics of metoclopramide in man with observations in the dog.* British Journal of Clinical Pharmacology, 1980. **9**(4): p. 371-377.

93. Bylsma-Howell, M., et al., *Placental transport of metoclopramide: assessment of maternal and neonatal effects.* Can Anaesth Soc J, 1983. **30**(5): p. 487-92.

94. Magueur, E., et al., *Pharmacokinetics of metoclopramide in patients with liver cirrhosis.* British Journal of Clinical Pharmacology, 1991. **31**(2): p. 185-187.

95. Ross-Lee, L.M., et al., *Single-dose pharmacokinetics of metoclopramide.* Eur J Clin Pharmacol, 1981. **20**(6): p. 465-71.

96. Chung, H., et al., *A non-linear pharmacokinetic-pharmacodynamic relationship of metformin in healthy volunteers: An open-label, parallel group, randomized clinical study.* PLOS ONE, 2018. **13**(1): p. e0191258.

97. Cullen, E., et al., *Pharmacokinetics and dose proportionality of extended-release metformin following administration of 1000, 1500, 2000 and 2500 mg in healthy volunteers.* Biopharm Drug Dispos, 2004. **25**(6): p. 261-3.

98. de Oliveira Baraldi, C., et al., *Metformin pharmacokinetics in nondiabetic pregnant women with polycystic ovary syndrome.* Eur J Clin Pharmacol, 2011. **67**(10): p. 1027-33.

99. Eyal, S., et al., *Pharmacokinetics of metformin during pregnancy.* Drug Metab Dispos, 2010. **38**(5): p. 833-40.

100. Lee, S.H. and K.-i. Kwon, *Pharmacokinetic-pharmacodynamic modeling for the relationship between glucose-lowering effect and plasma concentration of metformin in volunteers.* Archives of Pharmacal Research, 2004. **27**(7): p. 806-810.

101. Liao, M.Z., et al., *Effects of Pregnancy on the Pharmacokinetics of Metformin.* Drug Metab Dispos, 2020. **48**(4): p. 264-271.

102. SAMBOL, N.C., et al., *Food intake and dosage level, but not tablet vs solution dosage form, affect the absorption of metformin HC1 in man.* British Journal of Clinical Pharmacology, 1996. **42**(4): p. 510-512.

103. Sirtori, C.R., et al., *Disposition of metformin (N,N-dimethylbiguanide) in man.* Clinical Pharmacology & Therapeutics, 1978. **24**(6): p. 683-693.

104. Tertti, K., et al., *The degree of fetal metformin exposure does not influence fetal outcome in gestational diabetes mellitus.* Acta Diabetologica, 2014. **51**(5): p. 731-738.

105. Tucker, G., et al., *Metformin kinetics in healthy subjects and in patients with diabetes mellitus.* British Journal of Clinical Pharmacology, 1981. **12**(2): p. 235-246.

106. Creinin, M.D. and M.A. Krohn, *Methotrexate pharmacokinetics and effects in women receiving methotrexate 50 mg and 60 mg per square meter for early abortion.* American Journal of Obstetrics and Gynecology, 1997. **177**(6): p. 1444-1449.

107. Hoekstra, M., et al., *Bioavailability of higher dose methotrexate comparing oral and subcutaneous administration in patients with rheumatoid arthritis.* J Rheumatol, 2004. **31**(4): p. 645-8.

108. Kozloski, G.D., et al., *The effect of food on the absorption of methotrexate sodium tablets in healthy volunteers.* Arthritis Rheum, 1992. **35**(7): p. 761-4.

109. Pichlmeier, U. and K.U. Heuer, *Subcutaneous administration of methotrexate with a prefilled autoinjector pen results in a higher relative bioavailability compared with oral administration of methotrexate.* Clin Exp Rheumatol, 2014. **32**(4): p. 563-71.

110. Schornagel, J.H., M.E. Van Engelen, and D. De Vos, *Bioavailability of methotrexate tablets.* Pharm Weekbl Sci, 1982. **4**(3): p. 89-90.

111. Yamazaki, T., et al., *Pharmacokinetic Interactions Between Isavuconazole and the Drug Transporter Substrates Atorvastatin, Digoxin, Metformin, and Methotrexate in Healthy Subjects.* Clin Pharmacol Drug Dev, 2017. **6**(1): p. 66-75.

112. Zuo, X., X. Zhao, and T. Zhang, *Pharmacokinetics and bioequivalence evaluation of 2 oral formulations of methotrexate tablets in healthy Chinese volunteers under fasting and fed conditions.* Naunyn Schmiedebergs Arch Pharmacol, 2023. **396**(4): p. 803-809.

113. Chan, K.K., et al., *Clinical pharmacokinetics of cyclophosphamide and metabolites with and without SR-2508.* Cancer Res, 1994. **54**(24): p. 6421-9.

114. D'Incalci, M., et al., *Transplacental passage of cyclophosphamide.* Cancer Treat Rep, 1982. **66**(8): p. 1681-2.

115. De Bruijn, E.A., et al., *Pharmacokinėtics of intravenous and oral cyclophosphamide in the presence of methotrexate and fluorouracil.* Pharmaceutisch Weekblad, 1988. **10**(5): p. 200-206.

116. Juma, F.D., et al., *Pharmacokinetics of intravenous cyclophosphamide in man, estimated by gas-liquid chromatography.* Cancer Chemotherapy and Pharmacology, 1978. **1**(4): p. 229-231.

117. Stewart, D.J., et al., *Pharmacology, relative bioavailability, and toxicity of three different oral cyclophosphamide preparations in a randomized, cross-over study.* Invest New Drugs, 1995. **13**(1): p. 99-107.

118. Tchekmedyian, N.S., et al., *Phase I clinical and pharmacokinetic study of cyclophosphamide administered by five-day continuous intravenous infusion.* Cancer Chemotherapy and Pharmacology, 1986. **18**(1): p. 33-38.

119. Benedek, I.H., et al., *Effect of moricizine on the pharmacokinetics and pharmacodynamics of warfarin in healthy volunteers.* J Clin Pharmacol, 1992. **32**(6): p. 558-63.

120. King, S.Y., et al., *Dose-dependent pharmacokinetics of warfarin in healthy volunteers.* Pharm Res, 1995. **12**(12): p. 1874-7.

121. Ngo, N., et al., *The warfarin-cranberry juice interaction revisited: A systematic in vitro-in vivo evaluation.* J Exp Pharmacol, 2010. **2010**(2): p. 83-91.

122. Orme, M.L., et al., *May mothers given warfarin breast-feed their infants?* Br Med J, 1977. **1**(6076): p. 1564-5.

123. Toon, S., et al., *Investigations into the potential effects of multiple dose ketorolac on the pharmacokinetics and pharmacodynamics of racemic warfarin.* British Journal of Clinical Pharmacology, 1990. **30**(5): p. 743-750.

124. Bai, N., et al., *Determination of thalidomide concentration in human plasma by liquid chromatography-tandem mass spectrometry.* Exp Ther Med, 2013. **5**(2): p. 626-630.

125. Eriksson, T., et al., *Stereospecific determination, chiral inversion in vitro and pharmacokinetics in humans of the enantiomers of thalidomide.* Chirality, 1995. **7**(1): p. 44-52.

126. Piscitelli, S.C., et al., *Single-dose pharmacokinetics of thalidomide in human immunodeficiency virus-infected patients.* Antimicrobial Agents and Chemotherapy, 1997. **41**(12): p. 2797-2799.

127. Teo, S.K., W.A. Colburn, and S.D. Thomas, *Single-Dose Oral Pharmacokinetics of Three Formulations of Thalidomide in Healthy Male Volunteers.* The Journal of Clinical Pharmacology, 1999. **39**(11): p. 1162-1168.

128. Teo, S.K., et al., *Effect of a high-fat meal on thalidomide pharmacokinetics and the relative bioavailability of oral formulations in healthy men and women.* Biopharm Drug Dispos, 2000. **21**(1): p. 33-40.

129. Teo, S.K., et al., *Thalidomide dose proportionality assessment following single doses to healthy subjects.* J Clin Pharmacol, 2001. **41**(6): p. 662-7.

130. Matta, M.K., et al., *Effect of Sunscreen Application on Plasma Concentration of Sunscreen Active Ingredients: A Randomized Clinical Trial.* JAMA, 2020. **323**(3): p. 256-267.

131. Tarazona, I., A. Chisvert, and A. Salvador, *Determination of benzophenone-3 and its main metabolites in human serum by dispersive liquid–liquid microextraction followed by liquid chromatography tandem mass spectrometry.* Talanta, 2013. **116**: p. 388-395.

132. Kemp, H.A., et al., *Measurement of diethylstilbestrol in plasma from patients with cancer of the prostate.* Cancer Res, 1981. **41**(11 Pt 1): p. 4693-7.

133. Nakamura, K., *Bioavailability, distribution and pharmacokinetics of diethylstilbestrol converted from diethylstilbestrol diphosphate in patients with prostatic cancer.* Hiroshima J Med Sci, 1986. **35**(4): p. 325-38.

134. Zhang, H., et al., *Pharmacokinetics and safety profiles of novel diethylstilbestrol orally dissolving film in comparison with diethylstilbestrol capsules in healthy Chinese male subjects.* Int J Clin Pharmacol Ther, 2014. **52**(5): p. 407-15.

135. EGERMAN, R.S., et al., *A Comparison of the Bioavailability of Oral and Intramuscualr Dexamethasone in Women in Late Pregnancy.* Obstetrics & Gynecology, 1997. **89**(2): p. 276-280.

136. Elliott, C.L., G.F. Read, and E.M. Wallace, *The pharmacokinetics of oral and intramuscular administration of dexamethasone in late pregnancy.* Acta Obstet Gynecol Scand, 1996. **75**(3): p. 213-6.

137. Jobe, A.H., et al., *Pharmacokinetics and Pharmacodynamics of Intramuscular and Oral Betamethasone and Dexamethasone in Reproductive Age Women in India.* Clin Transl Sci, 2020. **13**(2): p. 391-399.

138. Loew, D., O. Schuster, and E.H. Graul, *Dose-dependent pharmacokinetics of dexamethasone.* Eur J Clin Pharmacol, 1986. **30**(2): p. 225-30.

139. Queckenberg, C., et al., *Pharmacokinetics, Pharmacodynamics, and Comparative Bioavailability of Single, Oral 2-mg Doses of Dexamethasone Liquid and Tablet Formulations: A Randomized, Controlled, Crossover Study in Healthy Adult Volunteers.* Clinical Therapeutics, 2011. **33**(11): p. 1831-1841.

140. Tsuei, S.E., et al., *Disposition of synthetic glucocorticoids: II. Dexamethasone in parturient women.* Clinical Pharmacology & Therapeutics, 1980. **28**(1): p. 88-98.

141. Verhagen, H., et al., *Disposition of single oral doses of butylated hydroxytoluene in man and rat.* Food and Chemical Toxicology, 1989. **27**(12): p. 765-772.

142. Nikiforov, A.I. and T.G. Osimitz, *Analysis and interpretation of pharmacokinetic studies following DEET administration to rats, dogs, and humans.* Toxicology Research and Application, 2022. **6**: p. 23978473221117230.

143. Ason, B., F.A. Armah, and D.K. Essumang, *Characterization and quantification of endocrine disruptors in female menstrual blood samples.* Toxicology Reports, 2022. **9**: p. 1877-1882.

144. Högberg, J., et al., *Phthalate diesters and their metabolites in human breast milk, blood or serum, and urine as biomarkers of exposure in vulnerable populations.* Environ Health Perspect, 2008. **116**(3): p. 334-9.

145. Huang, Y., et al., *Phthalate levels in cord blood are associated with preterm delivery and fetal growth parameters in Chinese women.* PLoS One, 2014. **9**(2): p. e87430.

146. Reddy, B.S., et al., *Association of phthalate esters with endometriosis in Indian women.* Bjog, 2006. **113**(5): p. 515-20.

147. Rozati, R., et al., *Evaluation of the Phthalate Esters in South Indian Women with Endometriosis.* International Journal of Fertility and Sterility, 2008. **1**: p. 165-170.

148. Zhang, S.-h., et al., *Phthalate exposure and high blood pressure in adults: a cross-sectional study in China.* Environmental Science and Pollution Research, 2018. **25**(16): p. 15934-15942.

149. Barr, D.B., et al., *Pesticide concentrations in maternal and umbilical cord sera and their relation to birth outcomes in a population of pregnant women and newborns in New Jersey.* Science of The Total Environment, 2010. **408**(4): p. 790-795.

150. Liao, H.-T., et al., *Simultaneous analysis of chlorpyrifos and cypermethrin in cord blood plasma by online solid-phase extraction coupled with liquid chromatography–heated electrospray ionization tandem mass spectrometry.* Journal of Chromatography B, 2011. **879**(21): p. 1961-1966.

151. Perera, F.P., et al., *Effects of transplacental exposure to environmental pollutants on birth outcomes in a multiethnic population.* Environ Health Perspect, 2003. **111**(2): p. 201-5.

152. Whyatt, R.M., et al., *Contemporary-use pesticides in personal air samples during pregnancy and blood samples at delivery among urban minority mothers and newborns.* Environ Health Perspect, 2003. **111**(5): p. 749-56.
