# Supplementary material for: An advancement in developmental and reproductive toxicity (DART) risk assessment: evaluation of a bioactivity and exposure-based NAM toolbox
Source: Front Toxicol. 2025 Jun 30;7:1602065. doi: 10.3389/ftox.2025.1602065 (PMC12256496; doi:10.3389/ftox.2025.1602065)
Supplement: Supplementary file 4 [file DataSheet2.docx]

**Evaluation of in silico models**

**Selection of models**

Preliminary, 14 models predicting DART and DART relevant activities within four platforms namely Derek Nexus v. 6.2.0 (Marchant CA, et al.), OECD QSAR Toolbox (https://qsartoolbox.org/), VEGA (Benfenati E, 2013) and Open (Quantitative) Structure-activity/property Relationship App (OPERA) v.2.8 (Mansouri et al., 2016) have been selected. Table S1 provides information about the 14 selected models. Five models are predicting general DART toxicity, and nine models are mode of action (MoA) specific models predicting estrogen, androgen and thyroid receptor effects. Some models like CERAPP and CoMPARA or models based on P&G decision tree (Wu et al., 2013) are present in two platforms/tools. The reason for the duplication is the differences between the models. DART P&G scheme in OECD QSAR Toolbox is based on original Wu et al paper and provides only the category, whereas the VEGA_DEVTOX_PG model calculates not only the predictive assessment, but also the most similar compounds of the training set, an applicability domain index (ADI) and based on this, indicates a reliability (QRMFa). The specific models, CoMPARA and CERAPP (Mansouri et al., 2016, Mansouri et al., 2020) present in OPERA platform, predict binding as well as agonist and antagonist activity, together with the applicability domain (global and local), confidence score and nearest neighbours. The same models in VEGA platform give the assessment of binding affinity with ADI and reliability (QRMFb, QRMFc). Derek Nexus is the expert knowledge base system, and it is providing structural alerts for more than 70 different endpoints. However, for the evaluation of predictiveness for DART related effects, two subsets of endpoints have been considered: one with 17 most relevant DART endpoints and the second subsets with 34 endpoints most relevant for DART and systemic toxicity (see main text Table2).

Table S1. Information on in silico models

| **ID** | **Model** | **Tool/Platform** | **Endpoint** | **Method** | **Open source** | **Link to tool** |
| --- | --- | --- | --- | --- | --- | --- |
| 1 | Derek Nexus (17 endpoints) | Derek Nexus | DART general | Rule based / expert knowledge | NO | https://www.lhasalimited.org/solutions/ |
| 2 | Derek Nexus (34 endpoints) | Derek Nexus | DART general | Rule based / expert knowledge | NO | https://www.lhasalimited.org/solutions/ |
| 3 | DART Scheme | OECD QSAR Toolbox | DART general | Rule based | YES | https://qsartoolbox.org/ |
| 4 | Estrogen Receptor Binding | OECD QSAR Toolbox | MoA- Estrogen Receptor effect | Rule based | YES | https://qsartoolbox.org/ |
| 5 | rtER Expert System - USEPA | OECD QSAR Toolbox | MoA- Estrogen Receptor effect | Rule based | YES | https://qsartoolbox.org/ |
| 6 | DEVTOX_PG | VEGA | DART general | Rule based | YES | https://www.vegahub.eu/ |
| 7 | DevTox_CAESAR | VEGA | DART general | QSAR | YES | https://www.vegahub.eu/ |
| 8 | Estrogen Receptor Relative Binding Affinity model (IRFMN) | VEGA | MoA- Estrogen Receptor effect | QSAR | YES | https://www.vegahub.eu/ |
| 9 | ESTROGEN_CERAPP | VEGA | MoA- Estrogen Receptor effect | QSAR | YES | https://www.vegahub.eu/ |
| 10 | ANDROGEN_COMPARA | VEGA | MoA - Androgen Receptor effect | QSAR | YES | https://www.vegahub.eu/ |
| 11 | Thyroid Receptor Alpha effect (NRMEA) | VEGA | MoA - Thyroid Receptor effect | Rule based | YES | https://www.vegahub.eu/ |
| 12 | Thyroid Receptor Beta effect (NRMEA) | VEGA | MoA - Thyroid Receptor effect | Rule based | YES | https://www.vegahub.eu/ |
| 13 | CERAPP | OPERA | MoA- Estrogen Receptor effect | QSAR | YES | https://ntp.niehs.nih.gov/whatwestudy/niceatm/comptox/ct-opera/opera |
| 14 | CoMPAR | OPERA | MoA - Androgen Receptor effect | QSAR | YES | https://ntp.niehs.nih.gov/whatwestudy/niceatm/comptox/ct-opera/opera |

**Collation and curation of data used for the evaluation**

Experimental data from DART relevant studies have been collated from the literature and divided into three subsets: developmental in vivo (Challa et al., 2020, Ciallella et al., 2022), developmental in vitro ((Zurlinden et al., 2020); <https://toxys.com/reprotracker/>), and reproductive in vivo (Feng et al., 2021). After excluding overlapping chemicals and substances without discrete structures (e.g., mixtures), 1715 compounds were identified in the developmental in vivo data set, 1076 compounds in the developmental in vitro data set, and 1603 compounds in the reproductive in vivo data set. To ensure reliable evaluation, many of the chemicals known to be in the training sets of the selected 14 in silico models have been removed, this would include for example the removal of 637 chemicals in the developmental in vivo data set. After removing inorganics and metalorganics remaining structures have been desalted and neutralized. The final datasets ready for the evaluation of in silico models consist of 1017 chemicals in the developmental in vivo set, 1062 compounds in the developmental in vitro set, and 1376 compounds in the reproductive in vivo set. Both in vivo datasets are well balanced, with 462 positive and 555 negative compounds in developmental data set and 595 positive and 781 negative compounds in the reproductive data set. The developmental in vitro dataset is largely represented by negative chemicals (838) with only 224 positive compounds.

**The predictive performance of 14 in silico models**

The predictive performance of the preliminary selected models has been assessed within the different data sets (Table S2-S4) for sensitivity, specificity and accuracy (see also material and methods in main text). To simplify the interpretation of the results, only binary outputs of the selected models have been considered without additional predictions details, like reliability, likelihood, applicability domain, confidence score in this study. This was done as there is no defined way how to interpret the predictions with these additional information’s, which are very often model specific. Models were selected based on a balanced performance between specificity and sensitivity across different data sets, combined with deep expert knowledge and many years of working experience. Exception are MIE specific tools showing lower sensitivity with good Specificity as these are expected since many compounds show different molecular initiating events causing DART.

Table S2 Predictive performance for developmental in vivo dataset

| **Developmental\Teratogenecity in vivo 1017, 462 positive, 555 negative** | | | | | | | |
| --- | --- | --- | --- | --- | --- | --- | --- |
| Model | TP | FN | TN | FP | SE | SP | ACC |
| Derek Nexus (17 endpoints) | 195 | 267 | 410 | 145 | 0.42 | 0.74 | 0.59 |
| Derek Nexus (34 endpoints) | 350 | 112 | 214 | 341 | 0.76 | 0.39 | 0.55 |
| DART Scheme | 132 | 329 | 456 | 99 | 0.29 | 0.82 | 0.58 |
| Estrogen Receptor Binding (undifiened -2) | 48 | 414 | 515 | 40 | 0.10 | 0.93 | 0.55 |
| rtER Expert System - USEPA | 13 | 447 | 536 | 19 | 0.03 | 0.97 | 0.54 |
| VEGA_DEVTOX_PG | 169 | 293 | 419 | 136 | 0.37 | 0.75 | 0.58 |
| VEGA_DevTox_CAESAR | 359 | 103 | 267 | 288 | 0.78 | 0.48 | 0.62 |
| VEGA_Estrogen Receptor Relative Binding Affinity model (IRFMN) | 79 | 383 | 486 | 69 | 0.17 | 0.88 | 0.56 |
| VEGA_ESTROGEN_CERAPP | 24 | 394 | 435 | 31 | 0.05 | 0.78 | 0.45 |
| VEGA_ ANDROGEN_COMPARA | 66 | 396 | 529 | 26 | 0.14 | 0.95 | 0.59 |
| VEGA_Thyroid Receptor Alpha effect (NRMEA) | 0 | 462 | 553 | 2 | 0.00 | 1.00 | 0.54 |
| VEGA_Thyroid Receptor Beta effect (NRMEA) | 4 | 458 | 553 | 2 | 0.01 | 1.00 | 0.55 |
| OPERA_CERAPP | 62 | 400 | 500 | 55 | 0.13 | 0.90 | 0.55 |
| OPERA_CoMPAR | 196 | 266 | 428 | 127 | 0.42 | 0.77 | 0.61 |

TP – true positive, FN- false negative, TN - true negative, FP – false positive, SE- sensitivity = 𝑇𝑃/(𝑇𝑃+𝐹𝑁), SP – specificity = TN/(TN+FP), ACC – Accuracy = (TP+TN)/( 𝑇𝑃+𝑇𝑁+𝐹𝑃+𝐹𝑁)

Table S3 Predictive performance for developmental in vitro dataset

| Developmental in vitro 1062, 224 positive, 838 negative | | | | | | | |
| --- | --- | --- | --- | --- | --- | --- | --- |
| Model | TP | FN | TN | FP | SE | SP | ACC |
| Derek Nexus (17 endpoints) | 88 | 110 | 582 | 256 | 0.44 | 0.69 | 0.65 |
| Derek Nexus (34 endpoints) | 180 | 44 | 259 | 579 | 0.80 | 0.31 | 0.41 |
| DART Scheme | 90 | 108 | 580 | 258 | 0.45 | 0.69 | 0.65 |
| Estrogen Receptor Binding | 21 | 177 | 713 | 125 | 0.11 | 0.85 | 0.71 |
| rtER Expert System - USEPA | 21 | 177 | 742 | 94 | 0.11 | 0.89 | 0.74 |
| VEGA_DEVTOX_PG | 86 | 112 | 522 | 316 | 0.43 | 0.62 | 0.59 |
| VEGA_DevTox_CAESAR | 149 | 49 | 294 | 544 | 0.75 | 0.35 | 0.43 |
| VEGA_Estrogen Receptor Relative Binding Affinity model (IRFMN) | 45 | 153 | 720 | 118 | 0.23 | 0.86 | 0.74 |
| VEGA_ESTROGEN_CERAPP | 12 | 185 | 765 | 48 | 0.06 | 0.91 | 0.75 |
| VEGA_ ANDROGEN_COMPARA | 40 | 158 | 744 | 94 | 0.20 | 0.89 | 0.76 |
| VEGA_Thyroid Receptor Alpha effect (NRMEA) | 1 | 197 | 837 | 1 | 0.01 | 1.00 | 0.81 |
| VEGA_Thyroid Receptor Beta effect (NRMEA) | 0 | 198 | 835 | 3 | 0.00 | 1.00 | 0.81 |
| OPERA_CERAPP | 35 | 163 | 697 | 141 | 0.18 | 0.83 | 0.71 |
| OPERA_CoMPAR | 108 | 90 | 573 | 265 | 0.55 | 0.68 | 0.66 |

TP – true positive, FN- false negative, TN - true negative, FP – false positive, SE- sensitivity = 𝑇𝑃/(𝑇𝑃+𝐹𝑁), SP – specificity = TN/(TN+FP), ACC – Accuracy = (TP+TN)/( 𝑇𝑃+𝑇𝑁+𝐹𝑃+𝐹𝑁)

Table S4 Predictive performance for reproductive in vivo dataset

| **Reproductive in vivo 1376, 595 positive, 781 negative** | | | | | | | |
| --- | --- | --- | --- | --- | --- | --- | --- |
|  |  |  |  |  |  |  |  |
| Model | TP | FN | TN | FP | SE | SP | ACC |
| Derek Nexus (17 endpoints) | 305 | 288 | 653 | 128 | 0.51 | 0.84 | 0.70 |
| Derek Nexus (34 endpoints) | 441 | 152 | 402 | 379 | 0.74 | 0.51 | 0.61 |
| DARTScheme | 154 | 434 | 648 | 130 | 0.26 | 0.83 | 0.58 |
| Estrogen Receptor Binding | 119 | 471 | 735 | 44 | 0.20 | 0.94 | 0.62 |
| rtER Expert System - USEPA | 26 | 564 | 749 | 28 | 0.04 | 0.96 | 0.56 |
| VEGA_DEVTOX_PG | 278 | 315 | 577 | 204 | 0.47 | 0.74 | 0.62 |
| VEGA_DevTox_CAESAR | 451 | 142 | 350 | 431 | 0.76 | 0.45 | 0.58 |
| VEGA_Estrogen Receptor Relative Binding Affinity model (IRFMN) | 166 | 427 | 659 | 122 | 0.28 | 0.84 | 0.60 |
| VEGA_ESTROGEN_CERAPP | 105 | 444 | 588 | 27 | 0.18 | 0.75 | 0.50 |
| VEGA_ ANDROGEN_COMPARA | 150 | 443 | 751 | 30 | 0.25 | 0.96 | 0.65 |
| VEGA_Thyroid Receptor Alpha effect (NRMEA) | 1 | 592 | 781 | 0 | 0.00 | 1.00 | 0.57 |
| VEGA_Thyroid Receptor Beta effect (NRMEA) | 3 | 590 | 779 | 2 | 0.01 | 1.00 | 0.57 |
| OPERA_CERAPP | 209 | 386 | 676 | 105 | 0.35 | 0.87 | 0.64 |
| OPERA_CoMPAR | 338 | 257 | 617 | 164 | 0.57 | 0.79 | 0.69 |

TP – true positive, FN- false negative, TN - true negative, FP – false positive, SE- sensitivity = 𝑇𝑃/(𝑇𝑃+𝐹𝑁), SP – specificity = TN/(TN+FP), ACC – Accuracy = (TP+TN)/( 𝑇𝑃+𝑇𝑁+𝐹𝑃+𝐹𝑁)

Marchant CA, Briggs, KA, & Long A. (2008). In Silico Tools for Sharing Data and Knowledge on Toxicity and Metabolism: Derek for Windows, Meteor, and Vitic. Toxicology Mechanisms and Methods, 18(2–3), 177–187. <https://doi.org/10.1080/15376510701857320>

Mansouri K, Abdelaziz A, Rybacka A, Roncaglioni A, Tropsha A, Varnek A, et al. CERAPP: Collaborative Estrogen Receptor Activity Prediction Project. Environ Health Perspect. 2016;124(7):1023-33.

Challa AP, Beam AL, Shen M, Peryea T, Lavieri RR, Lippmann ES, et al. Machine learning on drug-specific data to predict small molecule teratogenicity. Reprod Toxicol. 2020;95:148-58.

Ciallella HL, Russo DP, Sharma S, Li Y, Sloter E, Sweet L, et al. Predicting Prenatal Developmental Toxicity Based On the Combination of Chemical Structures and Biological Data. Environ Sci Technol. 2022;56(9):5984-98.

Zurlinden TJ, Saili KS, Rush N, Kothiya P, Judson RS, Houck KA, et al. Profiling the ToxCast Library With a Pluripotent Human (H9) Stem Cell Line-Based Biomarker Assay for Developmental Toxicity. Toxicol Sci. 2020;174(2):189-209.

Feng H, Zhang L, Li S, Liu L, Yang T, Yang P, et al. Predicting the reproductive toxicity of chemicals using ensemble learning methods and molecular fingerprints. Toxicol Lett. 2021;340:4-14.

QMRFa Developmental/Reproductive Toxicity library (PG) (version 1.1.2) https://www.vegahub.eu/vegahub-dwn/qmrf/QMRF_DEVTOX_PG.pdf

QMRFb Androgen Receptor-mediated effect (IRFMN/COMPARA) (version1.0.1) https://www.vegahub.eu/vegahub-dwn/qmrf/QMRF_ANDROGEN_COMPARA.pdf

QMRFc Estrogen Receptor-mediated effect (IRFMN/CERAPP) (version 1.0.1) https://www.vegahub.eu/vegahub-dwn/qmrf/QMRF_ESTROGEN_CERAPP.pdf

Mansouri K, Abdelaziz A, Rybacka A, Roncaglioni A, Tropsha A, Varnek A, Zakharov A, Worth A, Richard AM, Grulke CM, Trisciuzzi D, Fourches D, Horvath D, Benfenati E, Muratov E, Wedebye EB, Grisoni F, Mangiatordi GF, Incisivo GM, Hong H, Ng HW, Tetko IV, Balabin I, Kancherla J, Shen J, Burton J, Nicklaus M, Cassotti M, Nikolov NG, Nicolotti O, Andersson PL, Zang Q, Politi R, Beger RD, Todeschini R, Huang R, Farag S, Rosenberg SA, Slavov S, Hu X, Judson RS. CERAPP: Collaborative Estrogen Receptor Activity Prediction Project. Environ Health Perspect. 2016, 124:1023–1033; http://dx.doi.org/10.1289/ehp.1510267

Mansouri K, Kleinstreuer N, R. Judson, A. Williams, I. Shah, AND A. Richard. CoMPARA: Collaborative Modeling Project for Androgen Receptor Activity. ENVIRONMENTAL HEALTH PERSPECTIVES. National Institute of Environmental Health Sciences (NIEHS), Research Triangle Park, NC, 128(2):27002, (2020). https://doi.org/10.1289/EHP5580

Wu S., Fisher, J., Naciff, J., Laufersweiler, M., Lester, C., Daston, G., & Blackburn K. Framework for identifying chemicals with structural features associated with the potential to act as developmental or reproductive toxicants. Chemical Research in Toxicology, 2013 26(12):1840–1861.

Benfenati E MA, Gini GC. VEGA-QSAR AI inside a platform for predictive toxicology. PAI@ AI* IA 1107:21–28. 2013

BENFENATI E, M. A., GINI GC 2013. VEGA-QSAR AI inside a platform for predictive toxicology. *PAI@ AI* IA 1107:21–28*.

CHALLA, A. P., BEAM, A. L., SHEN, M., PERYEA, T., LAVIERI, R. R., LIPPMANN, E. S. & ARONOFF, D. M. 2020. Machine learning on drug-specific data to predict small molecule teratogenicity. *Reprod Toxicol,* 95**,** 148-158.

CIALLELLA, H. L., RUSSO, D. P., SHARMA, S., LI, Y., SLOTER, E., SWEET, L., HUANG, H. & ZHU, H. 2022. Predicting Prenatal Developmental Toxicity Based On the Combination of Chemical Structures and Biological Data. *Environ Sci Technol,* 56**,** 5984-5998.

FENG, H., ZHANG, L., LI, S., LIU, L., YANG, T., YANG, P., ZHAO, J., ARKIN, I. T. & LIU, H. 2021. Predicting the reproductive toxicity of chemicals using ensemble learning methods and molecular fingerprints. *Toxicol Lett,* 340**,** 4-14.

MANSOURI, K., ABDELAZIZ, A., RYBACKA, A., RONCAGLIONI, A., TROPSHA, A., VARNEK, A., ZAKHAROV, A., WORTH, A., RICHARD, A. M., GRULKE, C. M., TRISCIUZZI, D., FOURCHES, D., HORVATH, D., BENFENATI, E., MURATOV, E., WEDEBYE, E. B., GRISONI, F., MANGIATORDI, G. F., INCISIVO, G. M., HONG, H., NG, H. W., TETKO, I. V., BALABIN, I., KANCHERLA, J., SHEN, J., BURTON, J., NICKLAUS, M., CASSOTTI, M., NIKOLOV, N. G., NICOLOTTI, O., ANDERSSON, P. L., ZANG, Q., POLITI, R., BEGER, R. D., TODESCHINI, R., HUANG, R., FARAG, S., ROSENBERG, S. A., SLAVOV, S., HU, X. & JUDSON, R. S. 2016. CERAPP: Collaborative Estrogen Receptor Activity Prediction Project. *Environ Health Perspect,* 124**,** 1023-33.

MANSOURI, K., KLEINSTREUER, N., ABDELAZIZ, A. M., ALBERGA, D., ALVES, V. M., ANDERSSON, P. L., ANDRADE, C. H., BAI, F., BALABIN, I., BALLABIO, D., BENFENATI, E., BHHATARAI, B., BOYER, S., CHEN, J., CONSONNI, V., FARAG, S., FOURCHES, D., GARCIA-SOSA, A. T., GRAMATICA, P., GRISONI, F., GRULKE, C. M., HONG, H., HORVATH, D., HU, X., HUANG, R., JELIAZKOVA, N., LI, J., LI, X., LIU, H., MANGANELLI, S., MANGIATORDI, G. F., MARAN, U., MARCOU, G., MARTIN, T., MURATOV, E., NGUYEN, D. T., NICOLOTTI, O., NIKOLOV, N. G., NORINDER, U., PAPA, E., PETITJEAN, M., PIIR, G., POGODIN, P., POROIKOV, V., QIAO, X., RICHARD, A. M., RONCAGLIONI, A., RUIZ, P., RUPAKHETI, C., SAKKIAH, S., SANGION, A., SCHRAMM, K. W., SELVARAJ, C., SHAH, I., SILD, S., SUN, L., TABOUREAU, O., TANG, Y., TETKO, I. V., TODESCHINI, R., TONG, W., TRISCIUZZI, D., TROPSHA, A., VAN DEN DRIESSCHE, G., VARNEK, A., WANG, Z., WEDEBYE, E. B., WILLIAMS, A. J., XIE, H., ZAKHAROV, A. V., ZHENG, Z. & JUDSON, R. S. 2020. CoMPARA: Collaborative Modeling Project for Androgen Receptor Activity. *Environ Health Perspect,* 128**,** 27002.

WU, S., FISHER, J., NACIFF, J., LAUFERSWEILER, M., LESTER, C., DASTON, G. & BLACKBURN, K. 2013. Framework for identifying chemicals with structural features associated with the potential to act as developmental or reproductive toxicants. *Chem Res Toxicol,* 26**,** 1840-61.

ZURLINDEN, T. J., SAILI, K. S., RUSH, N., KOTHIYA, P., JUDSON, R. S., HOUCK, K. A., HUNTER, E. S., BAKER, N. C., PALMER, J. A., THOMAS, R. S. & KNUDSEN, T. B. 2020. Profiling the ToxCast Library With a Pluripotent Human (H9) Stem Cell Line-Based Biomarker Assay for Developmental Toxicity. *Toxicol Sci,* 174**,** 189-209.
