# Supplementary material for: An advancement in developmental and reproductive toxicity (DART) risk assessment: evaluation of a bioactivity and exposure-based NAM toolbox
Source: Front Toxicol. 2025 Jun 30;7:1602065. doi: 10.3389/ftox.2025.1602065 (PMC12256496; doi:10.3389/ftox.2025.1602065)

Supplementary Image 1: Estimated bioactivity exposure ratios (BERs) for adult, pregnant and fetal exposure High risk scenarios (yellow), uncertain risk scenarios (black) and low risk scenarios (blue)**.** BERs are plotted for the different DART subpopulations on a log10 scale and a conceptual BER threshold is shown by vertical dotted line at BER=1. Shape of points refer to the source of the Cmax used to calculate each BER.

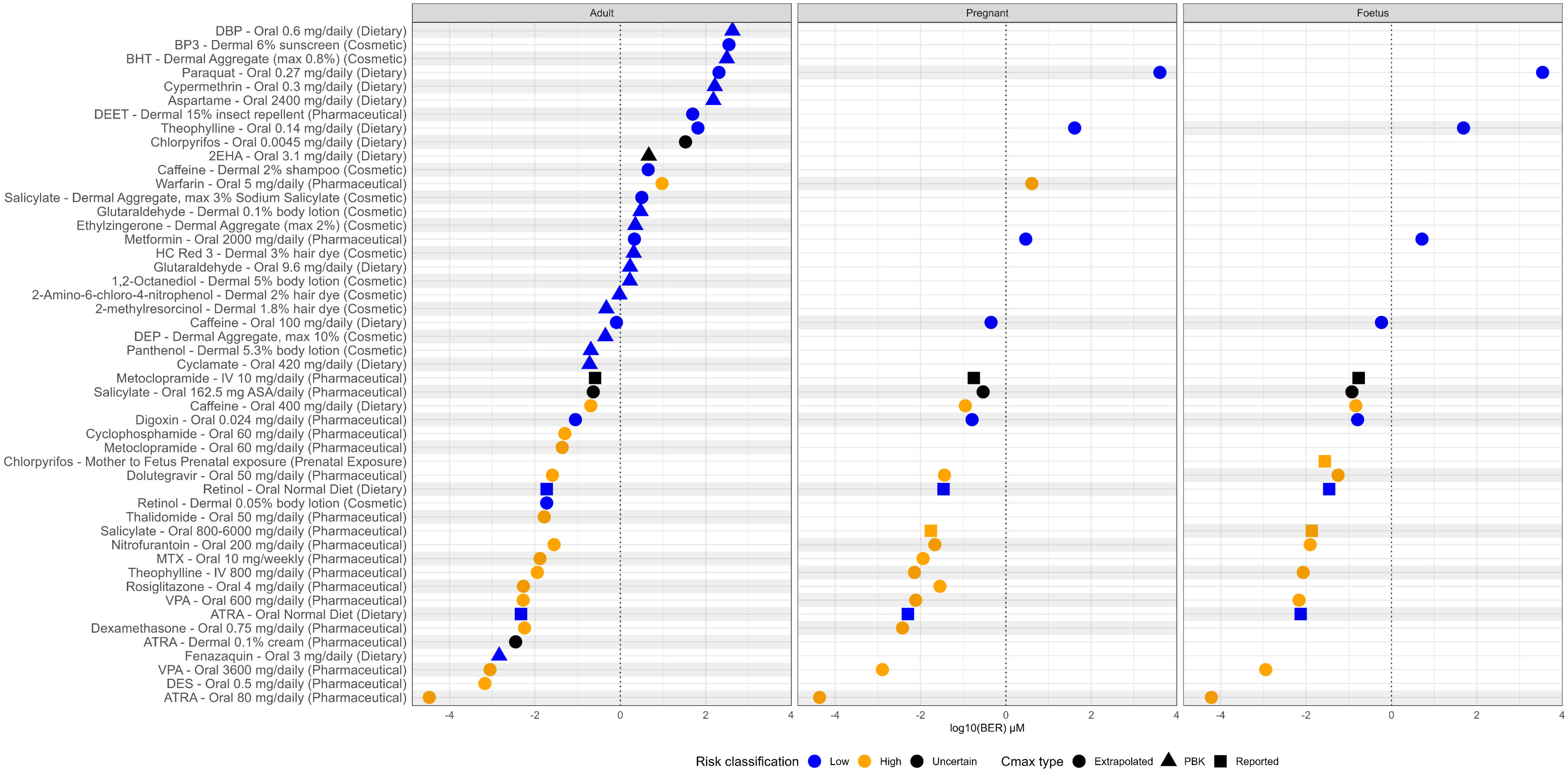

Supplement: Supplementary file 6 [file Image1.pdf]
